# Supplementary material for: Regulatory mechanism of cold-inducible diapause in Caenorhabditis elegans
Source: Nat Commun. 2024 Jul 10;15:5793. doi: 10.1038/s41467-024-50111-8 (PMC11237089; doi:10.1038/s41467-024-50111-8)
Supplement: Supplementary file 1 — Supplementary Information [file 41467_2024_50111_MOESM1_ESM.pdf]

Supplementary Information

Regulatory mechanism of cold-inducible diapause in *Caenorhabditis elegans*

Makoto Horikawa, Masamitsu Fukuyama, Adam Antebi, Masaki Mizunuma (2024)

Supplementary Figures 2-27
Supplementary Tables 28-36
Supplementary Methods 37-41

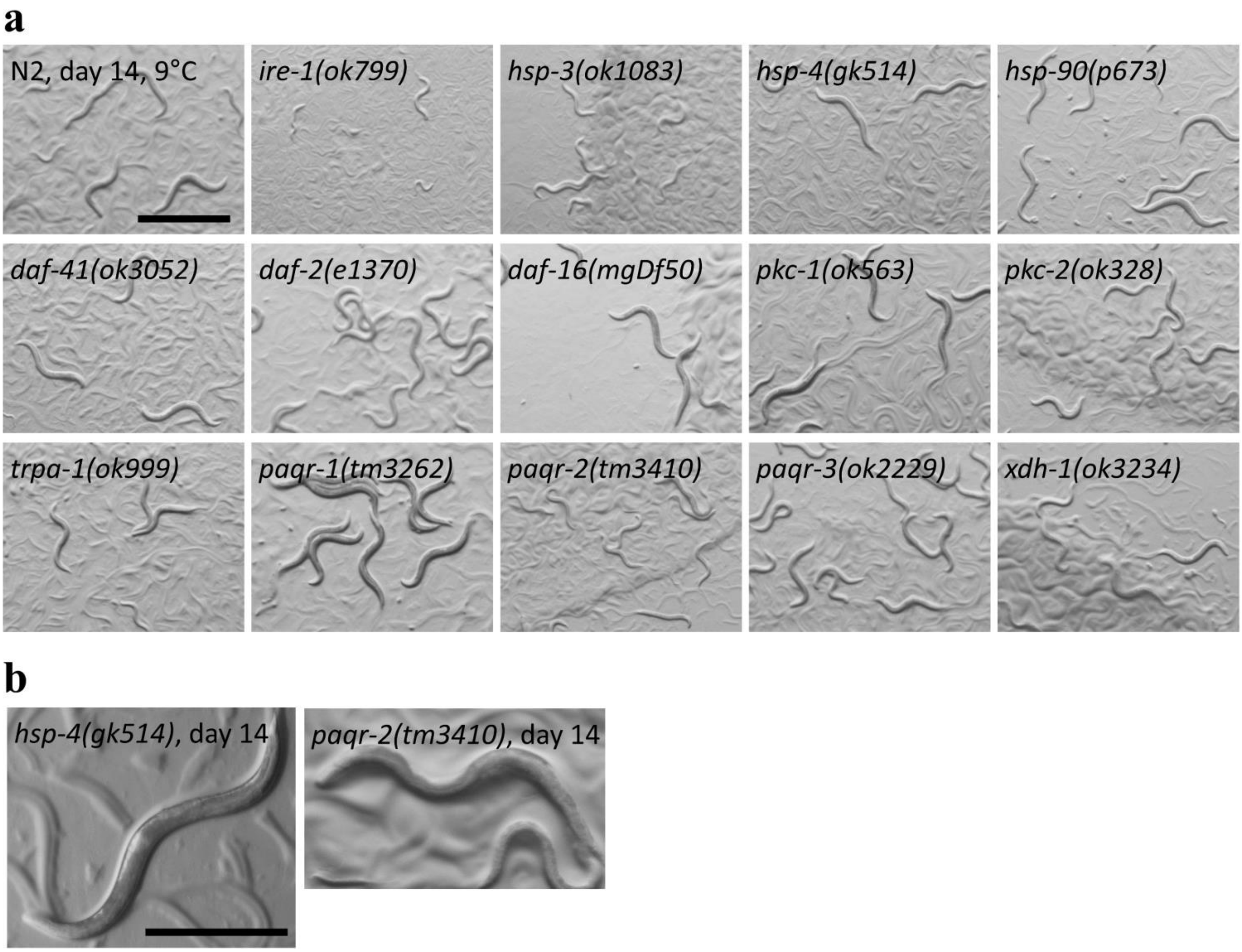

Supplementary Fig. 1 Phenotypic analysis of chaperone proteins and reported cold-sensitive mutants at 9 °C.

(**a**–**b**) UPR^{ER} mutants (*ire-1* and *hsp-3*) develop slower than wild-type animals at 9 °C (**a**) mutations in *hsp-4* and *paqr-2*, a nematode orthologue of the adiponectin receptor, cause adult sterility. (**b**) Other cold-sensitive mutants, *pkc-2(ok328), trpa-1(ok999), paqr-1(tm3262), paqr-3(ok2229),* and *xdh-1(ok3234)*, have no obvious cold sensitive phenotype at 9 °C. Scale bar, 1 mm (**a**) and 0.5 mm (**b**). n ≥ 3 biological replicates with more than 50 animals.

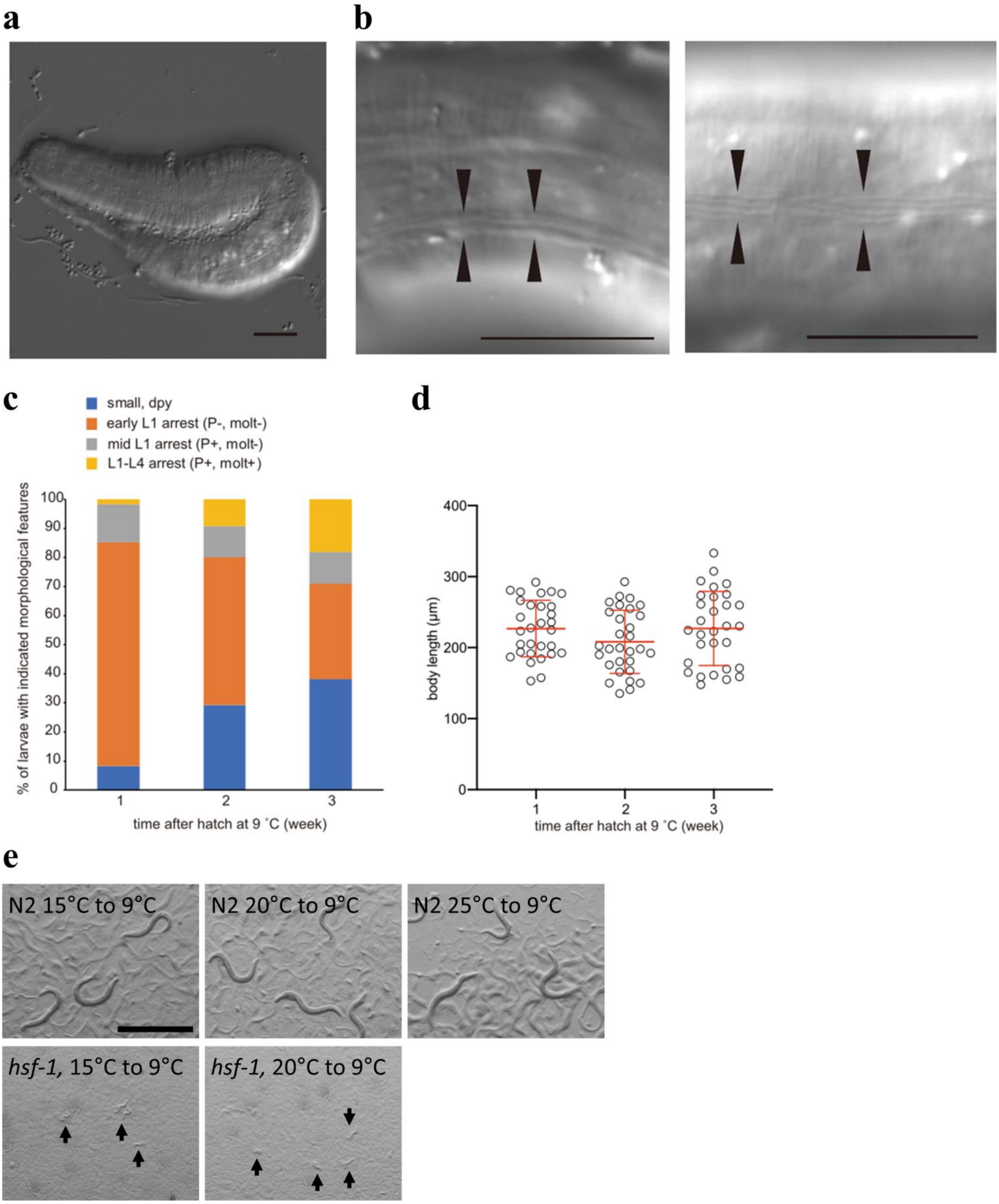

a
b
c
small, dpy
early L1 arrest (P-, molt-)
mid L1 arrest (P+, molt-)
L1-L4 arrest (P+, molt+)
% of larvae with indicated morphological features
time after hatch at 9 ˚C (week)
d
body length (µm)
time after hatch at 9 ˚C (week)
e
N2 15°C to 9°C
N2 20°C to 9°C
N2 25°C to 9°C
hsf-1, 15°C to 9°C
hsf-1, 20°C to 9°C

Supplementary Fig. 2 Morphological features of CID animals.

(a, b) Microscopic images of CID worms. An example of a small dumpy (dpy) arrested L1 larva. The animal was observed on day 21 (**a**). Examples of L1 alae of arrested animals. A few animals exhibited the canonical pattern of L1 alae (left). Other animals exhibited disorganised alae patterns (right). (**c**) The population of L1 arrested animals decreased slightly and that of dpy/dead animals increased with time. The experiments were repeated three times; 13–24 animals were scored at each time point for each experiment. At least 55 animals were scored in total at each time point. (**d**) The average sizes of the CID animals did not change on days 7, 14, and 21 of CID. (**e**) Maternal developmental temperatures did not affect CID formation in wild-type and *hsf-1(sy441)* mutants. The experiments were repeated three times and 10 animals were scored at each time point for each experiment (**a-d**). Scale bars, 10 μm (**a**, **b**), and 1 mm (**e**). $n \geq 3$ biological replicates with more than 50 animals (**e**). Arrows indicate arrested worms (**e**). Source data are provided as a Source Data file.

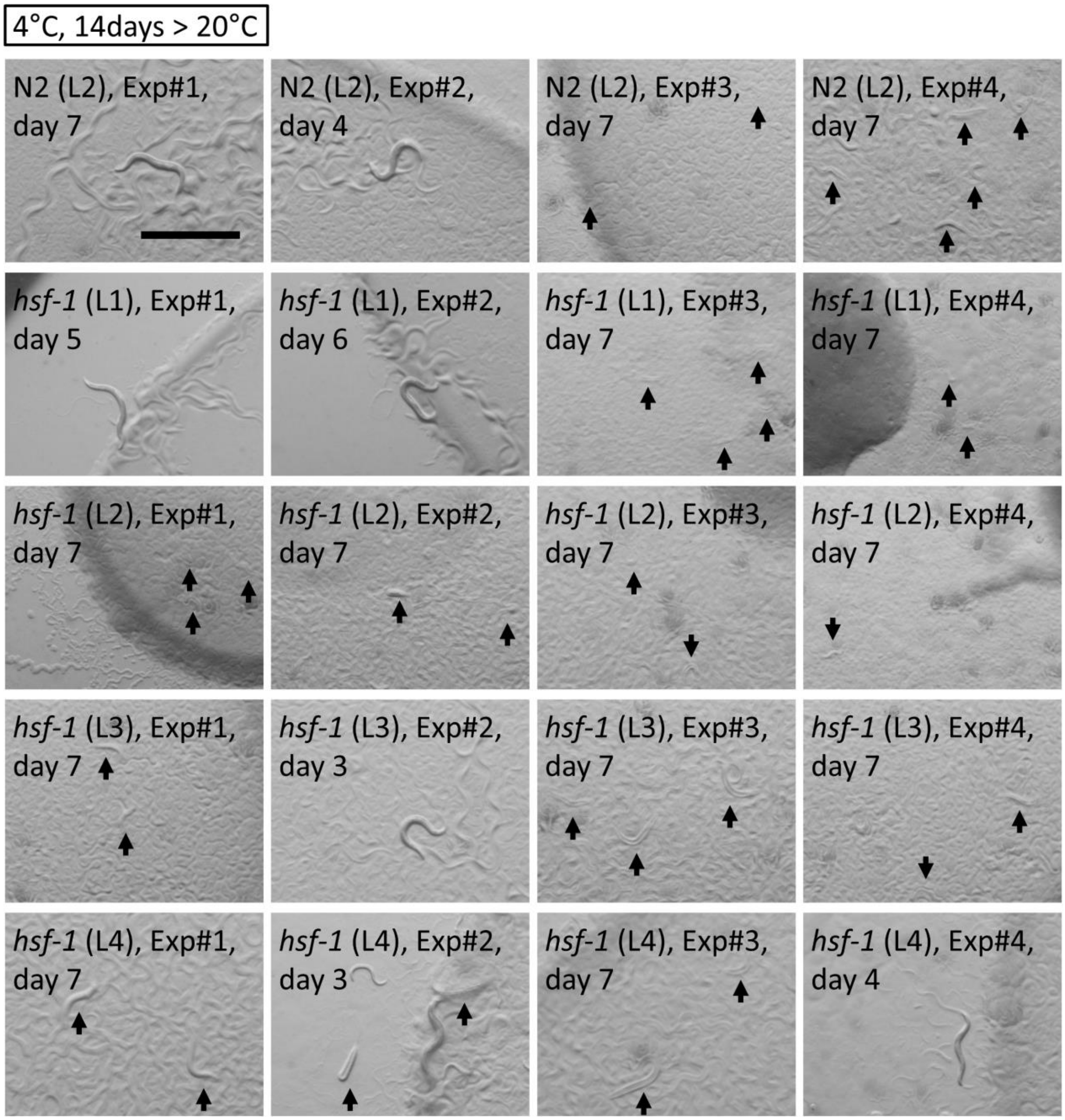

Supplementary Fig. 3 Recovery from CID induced at 4 °C.

CID was induced by a cold shift at 4 °C from L1, L2, L3 and L4 stages in wild-type animals and *hsf-1(sy441)* mutants and recovered at 20 °C after 14 days of cold exposure. Results of all four recovery experiments are presented. Scale bars, 1 mm. Arrows indicate dead worms.

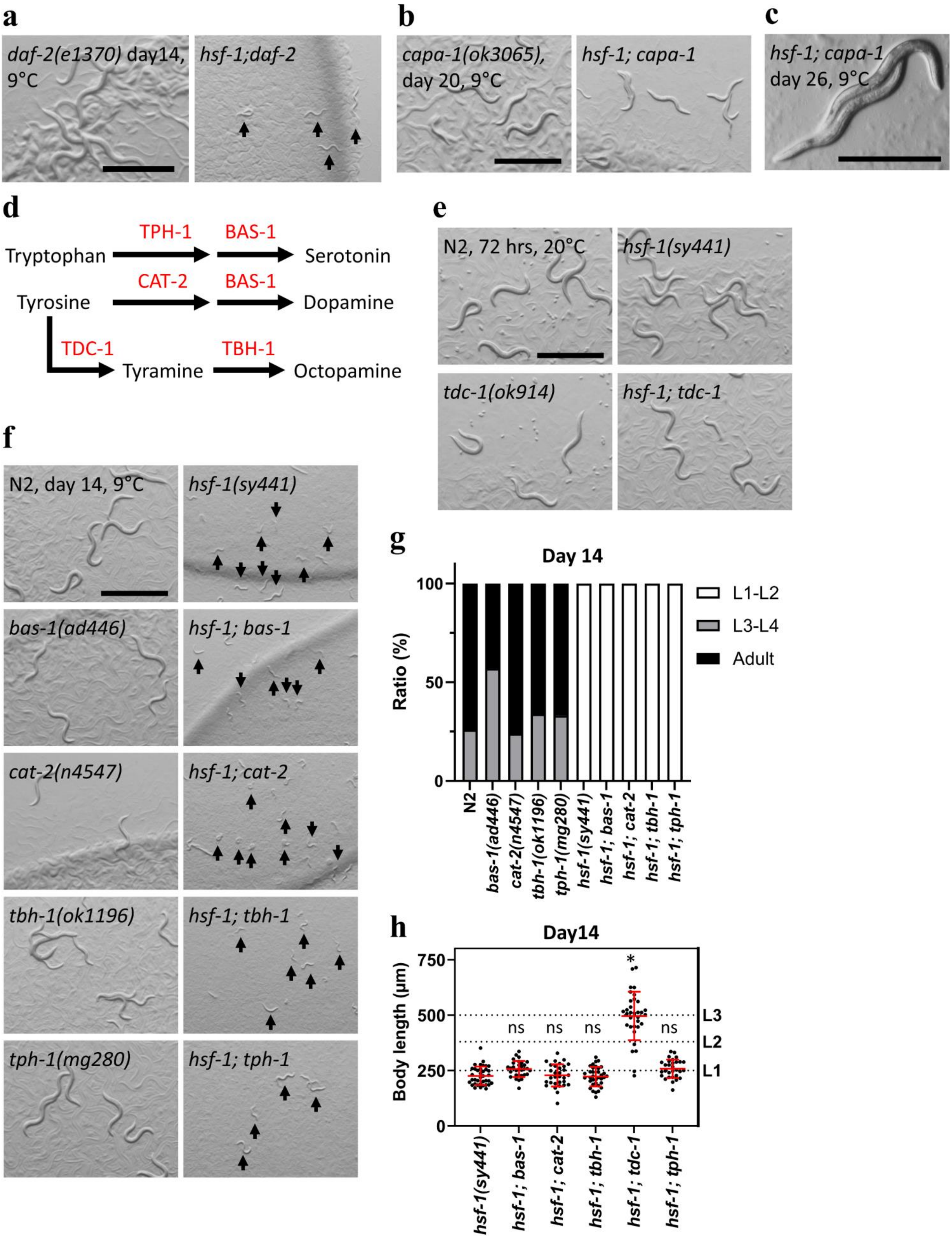
a
daf-2(e1370) day14, 9°C
hsf-1;daf-2
b
capa-1(ok3065), day 20, 9°C
hsf-1; capa-1
c
hsf-1; capa-1 day 26, 9°C
d
TPH-1
BAS-1
Tryptophan
Serotonin
CAT-2
BAS-1
Tyrosine
Dopamine
TDC-1
TBH-1
Tyramine
Octopamine
e
N2, 72 hrs, 20°C
hsf-1(sy441)
tdc-1(ok914)
hsf-1; tdc-1
f
N2, day 14, 9°C
hsf-1(sy441)
bas-1(ad446)
hsf-1; bas-1
cat-2(n4547)
hsf-1; cat-2
tbh-1(ok1196)
hsf-1; tbh-1
tph-1(mg280)
hsf-1; tph-1
g
Day 14
Ratio (%)
100
50
0
L1-L2
L3-L4
Adult
N2
bas-1(ad446)
cat-2(n4547)
tbh-1(ok1196)
tph-1(mg280)
hsf-1(sy441)
hsf-1; bas-1
hsf-1; cat-2
hsf-1; tbh-1
hsf-1; tph-1
h
Day14
Body length (μm)
750
500
250
0
L3
L2
L1
ns
*
hsf-1(sy441)
hsf-1; bas-1
hsf-1; cat-2
hsf-1; tbh-1
hsf-1; tdc-1
hsf-1; tph-1

Supplementary Fig. 4 Functions of insulin and monoamine signalling pathways in the regulation of *hsf-1(sy441*) mutant CID.

(a) A mutation in *daf-2* has no effect on CID entry. (**b, c)** The *capa-1(ok3065)* mutation caused weak and slow development at 9 °C. (**d)** A schematic illustration of monoamine neurotransmitter synthesis pathway in *C. elegans*[47]. (**e)** The *hsf-1; tdc-1* mutant developed normally in the same manner as did wild-type animals at 20 °C. (**f, g)** Mutations in monoamine neurotransmitter synthase enzymes did not inhibit CID entry at 9 °C. Although octopamine is synthesised from tyramine, a mutation in *tbh-1* did not rescue CID induction in the *hsf-1(sy441)* strain. White indicates the relative worm population of L1-L2 larvae, grey that of L3-L4 larvae and black that of adult worms. (**h)** The body length of the *hsf-1; tdc-1* mutant was larger than that of other mutants at 9 °C. Each dot represents the body length of a single worm. The red bars indicate the mean ± standard deviation (SD). The dashed lines indicate the maximum body length of each developmental stage. Asterisk indicates $P < 0.0001$ (vs *hsf-1(sy441)*) by ordinay one-way ANOVA. ns: Not significant. Scale bar, 0.5 mm (**c**), and 1 mm (**a, b, e, f**). $n \geq 3$ biological replicates with more than 50 animals. The graph represents one of the biological replicates (**g, h**). Arrows indicate arrested worms (**a, f**). Source data are provided as a Source Data file.

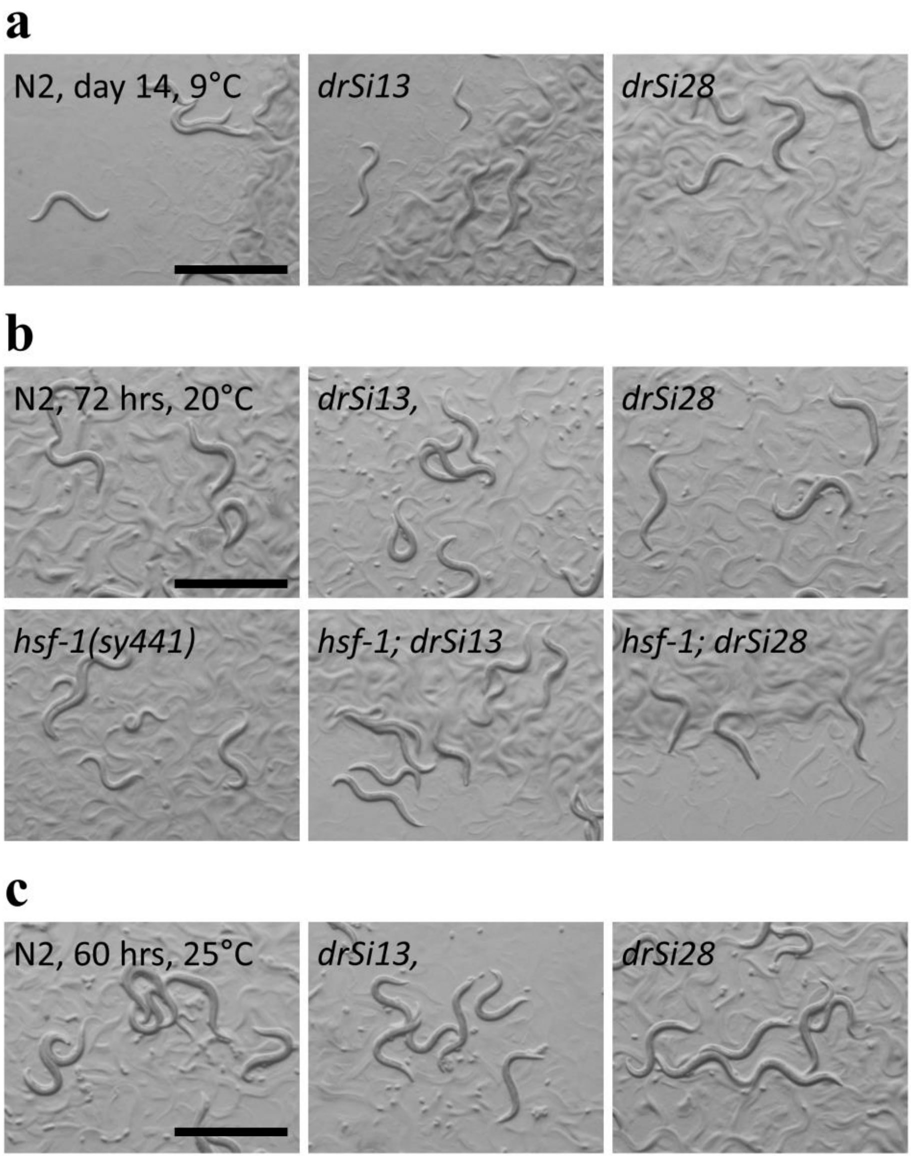

Supplementary Fig. 5 The *hsf-1* gene did not induce *hsf-1(sy441)* mutant CID and developmental arrest in wild-type animals.

(**a-c**) Transgenes of *drSi13[hsf-1p::hsf-1::gfp]* and *drSi28[hsf-1p::hsf-1(R145A)::gfp]* did not induc e CID at 9 °C and developmental arrest at 25 °C. Images were obtained at 60 h (**c**), 72 h (**b**), and on days 14 (**a**). (**a-c**) n ≥ 3 biological replicates with more than 50 animals. Scale bars, 1 mm.

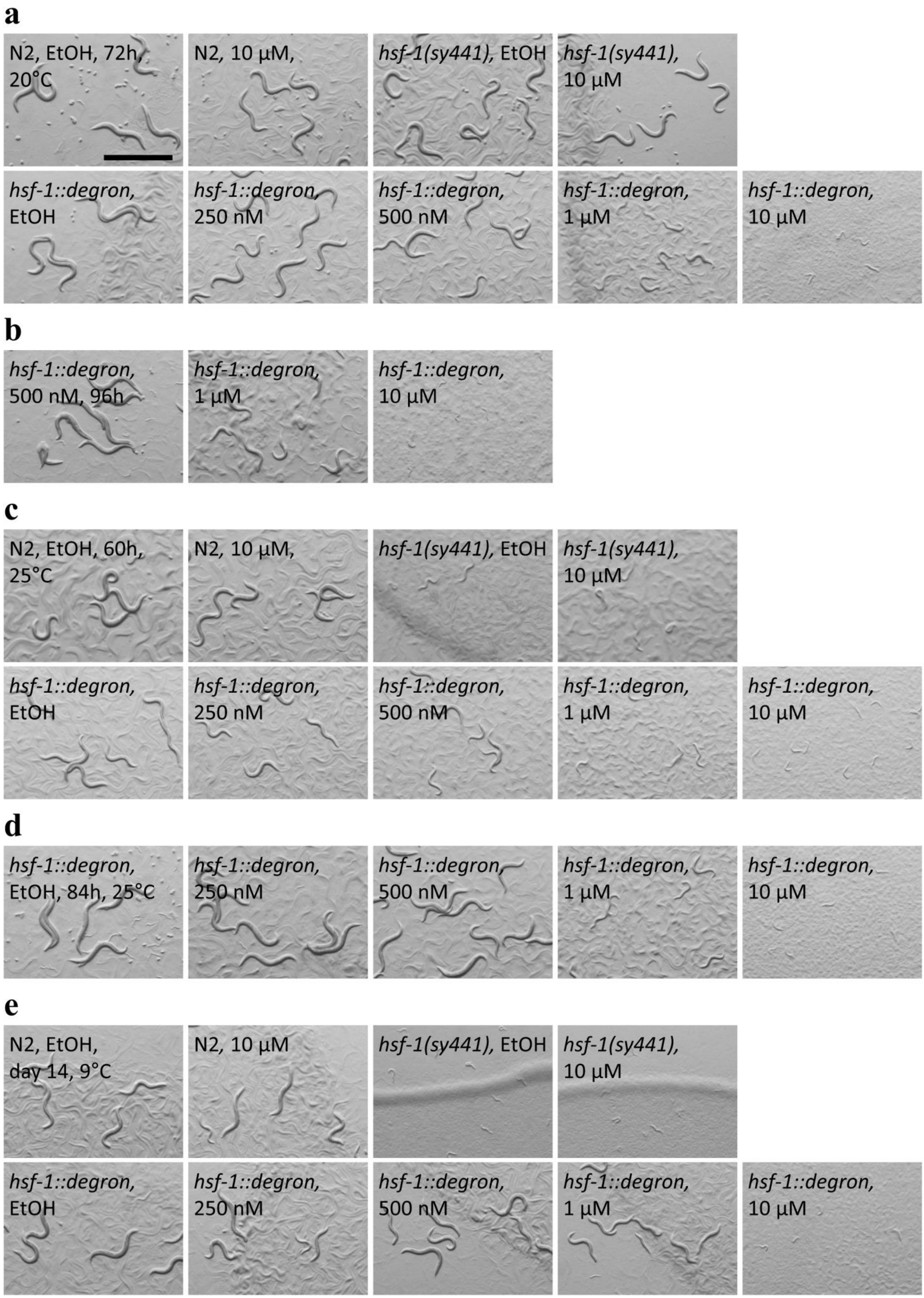
a
N2, EtOH, 72h, 20°C
N2, 10 µM,
hsf-1(sy441), EtOH
hsf-1(sy441), 10 µM
hsf-1::degron, EtOH
hsf-1::degron, 250 nM
hsf-1::degron, 500 nM
hsf-1::degron, 1 µM
hsf-1::degron, 10 µM
b
hsf-1::degron, 500 nM, 96h
hsf-1::degron, 1 µM
hsf-1::degron, 10 µM
c
N2, EtOH, 60h, 25°C
N2, 10 µM,
hsf-1(sy441), EtOH
hsf-1(sy441), 10 µM
hsf-1::degron, EtOH
hsf-1::degron, 250 nM
hsf-1::degron, 500 nM
hsf-1::degron, 1 µM
hsf-1::degron, 10 µM
d
hsf-1::degron, EtOH, 84h, 25°C
hsf-1::degron, 250 nM
hsf-1::degron, 500 nM
hsf-1::degron, 1 µM
hsf-1::degron, 10 µM
e
N2, EtOH, day 14, 9°C
N2, 10 µM
hsf-1(sy441), EtOH
hsf-1(sy441), 10 µM
hsf-1::degron, EtOH
hsf-1::degron, 250 nM
hsf-1::degron, 500 nM
hsf-1::degron, 1 µM
hsf-1::degron, 10 µM

Supplementary Fig. 6 Degradation of the HSF-1 protein by the AID system did not induce CID. **(a-b)** The threshold concentration of auxin inducing larval arrest at the L1 stage of *hsf-1(ljt3[hsf-1::degron::gfp]); ieSi57 [eft-3p::TIR1::mRuby]* worms was higher than 1 µM at 20 °C. (**c-d**) Partial suppression of HSF-1 protein with auxin treatment at 1 µM caused developmental arrest at 25°C (**e**) but did not induce CID at 9°C. Images were obtained at 60 h (**c**), 72h (**a**), 84 h (**d**), 96 h (**b**), and on days 14 (**e**). (**a-e**) n ≥ 3 biological replicates with more than 50 animals. Scale bars, 1 mm. Arrows indicate arrested worms.

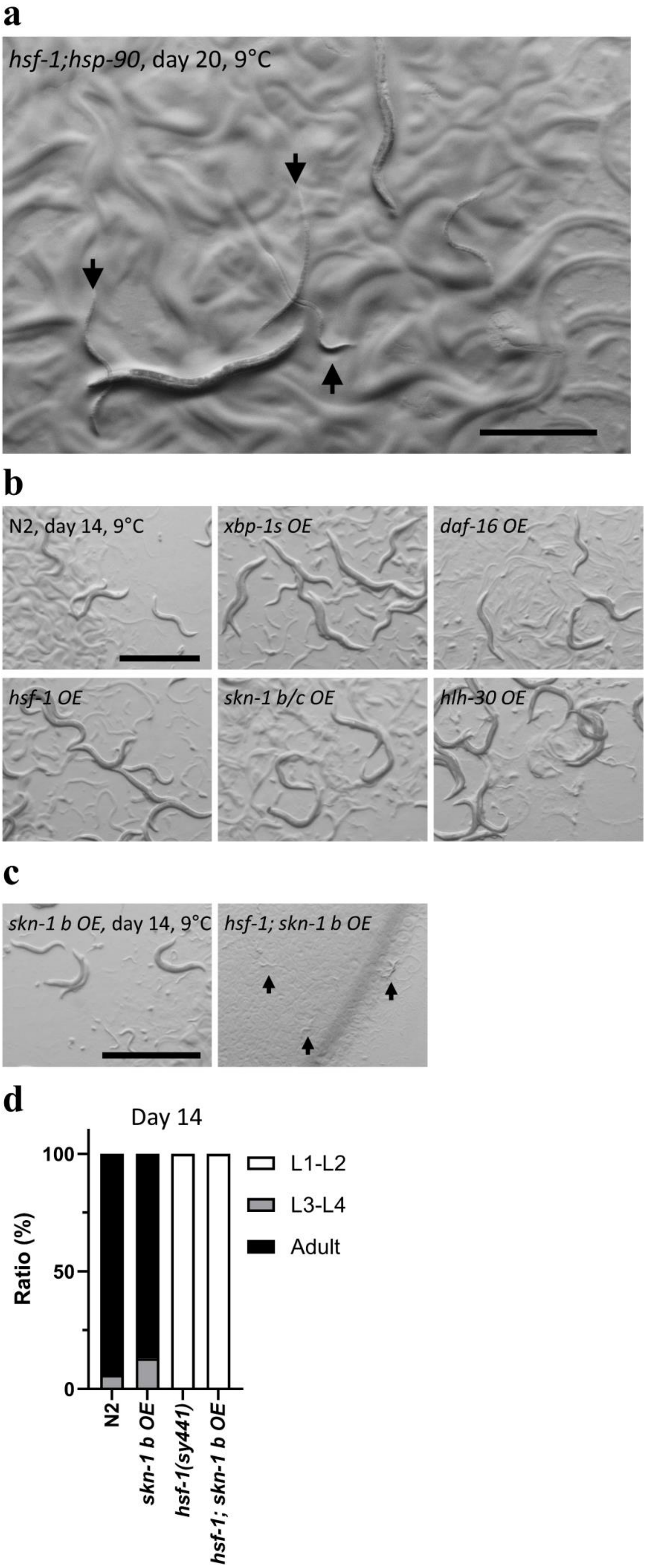
a
hsf-1;hsp-90, day 20, 9°C
b
N2, day 14, 9°C
xbp-1s OE
daf-16 OE
hsf-1 OE
skn-1 b/c OE
hlh-30 OE
c
skn-1 b OE, day 14, 9°C
hsf-1; skn-1 b OE
d
Day 14
Ratio (%)
100
50
0
L1-L2
L3-L4
Adult
N2
skn-1 b OE
hsf-1(sy441)
hsf-1; skn-1 b OE

Supplementary Fig. 7 Overexpression of anti-ageing genes did not induce CID in wild-type animals. (**a**) A gain-of-function mutation of *hsp-90* partially induced dauer at 9 °C. **(b**) Overexpression of anti-ageing genes, *xbp-1s* (active form), *skn-1 b/c*, *hlh-30*, and *daf-16,* did not induce CID entry in wild-ty pe animals. (**c-d**) Overexpression of neuronal isoform *skn-1b* did not inhibit CID formation of *hsf-1(s y441)* mutants. White indicates the relative worm population of L1-L2 larvae, grey that of L3-L4 larv ae and black that of adult worms. Scale bars, 1 mm. Images were obtained on days 14 (**b**, **c**) and days 20 (**a**). n ≥ 3 biological replicates with more than 50 animals. Graphs represent one of the biolo gical replicates. Arrows indicate dauer worms (**a**) and arrested worms (**c**). Source data are provi ded as a Source Data file.

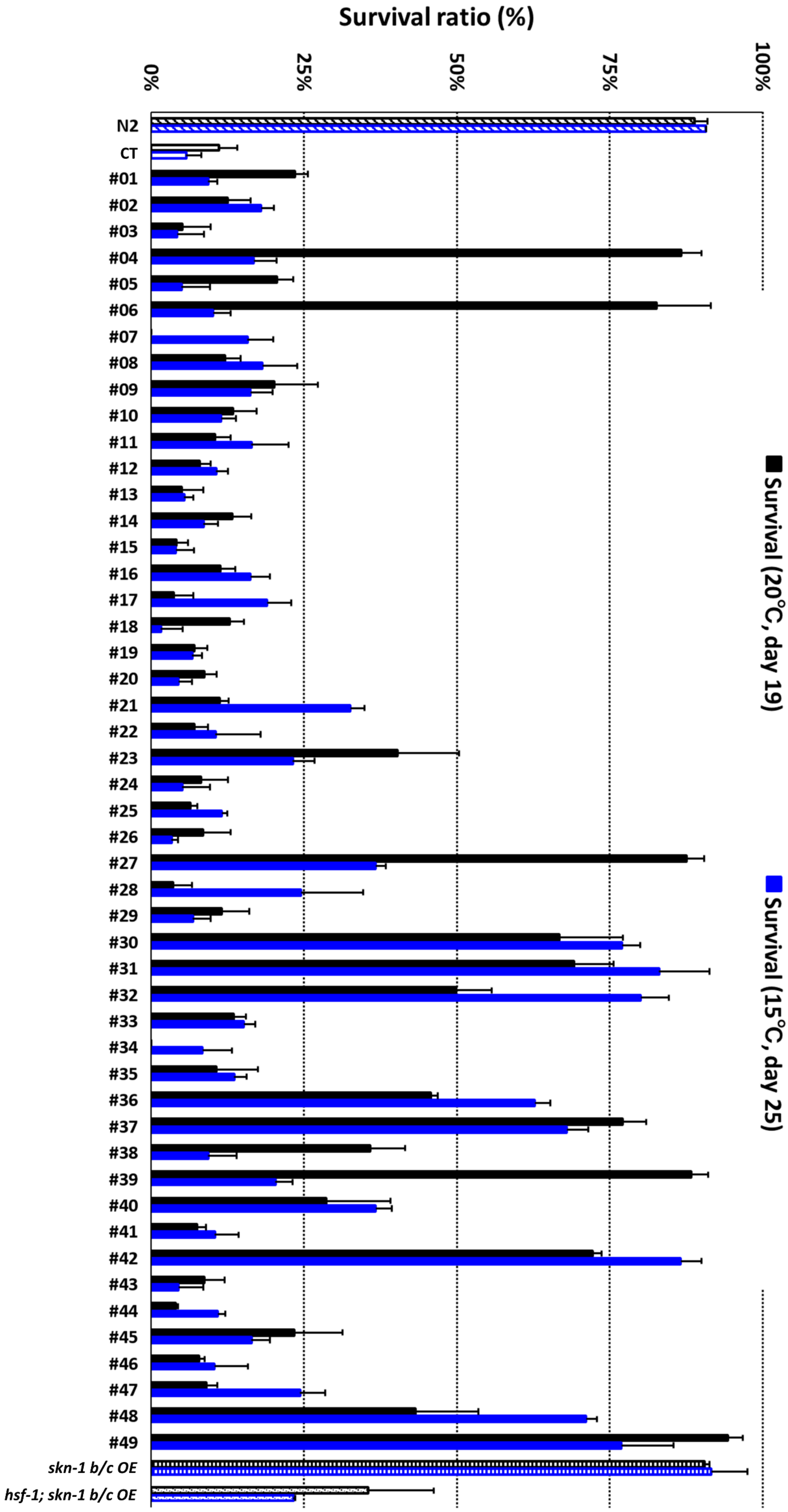
Survival ratio (%)
0%
25%
50%
75%
100%
Survival (20°C, day 19)
Survival (15°C, day 25)
N2
CT
#01
#02
#03
#04
#05
#06
#07
#08
#09
#10
#11
#12
#13
#14
#15
#16
#17
#18
#19
#20
#21
#22
#23
#24
#25
#26
#27
#28
#29
#30
#31
#32
#33
#34
#35
#36
#37
#38
#39
#40
#41
#42
#43
#44
#45
#46
#47
#48
#49
skn-1 b/c OE
hsf-1; skn-1 b/c OE

Supplementary Fig. 8 Survival profiles of non-CID mutants.

The graph represents the survival ratio of all non-CID mutant strains scored on day 19 at 15 °C and day 25 at 20 °C. Each technical replicate was scored for survival with more than 50 animals, and the experiment was performed once with three technical replicates. The bars indicate the mean of the survival rates ± standard deviation (SD). Source data are provided as a Source Data file.

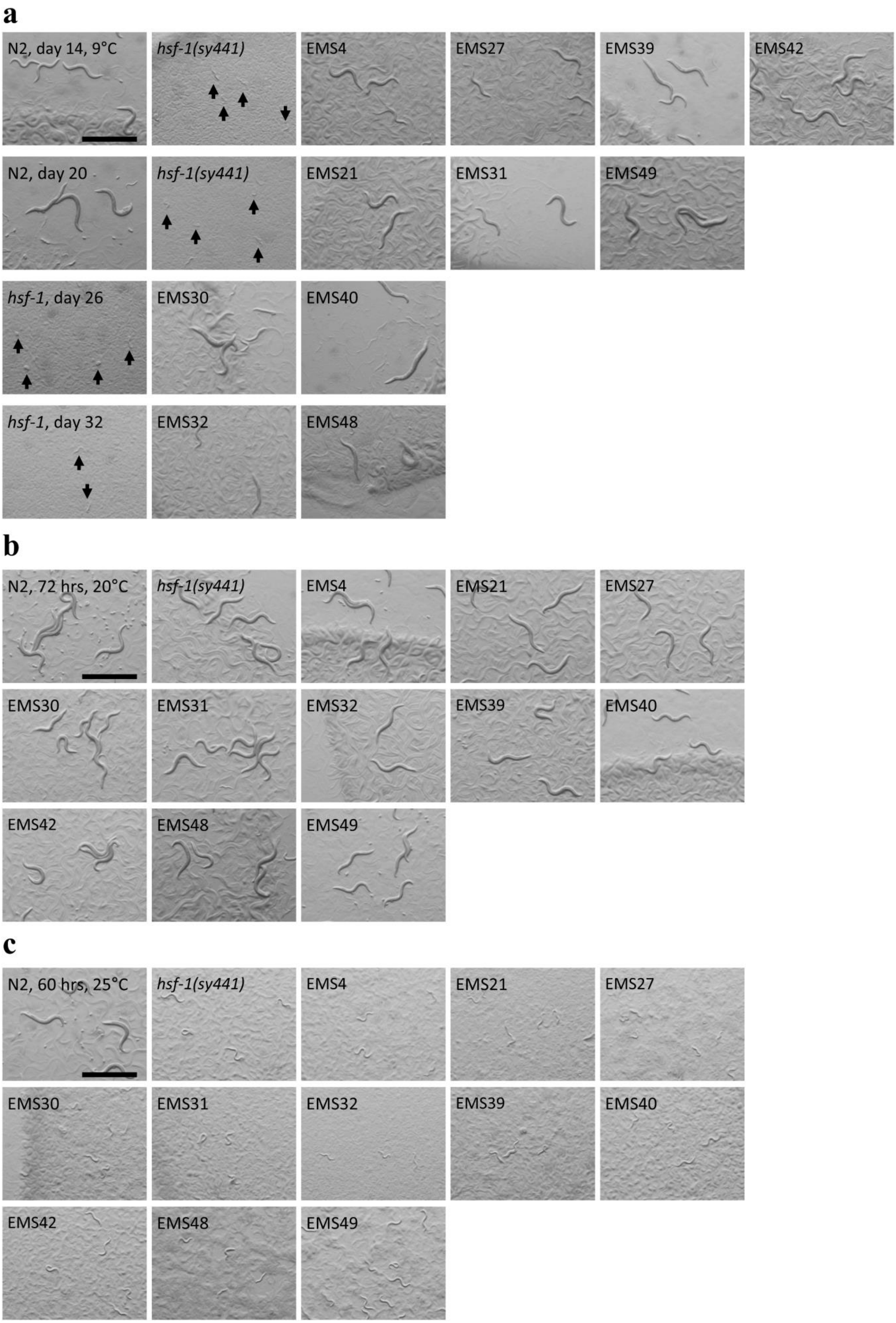
a
N2, day 14, 9°C
hsf-1(sy441)
EMS4
EMS27
EMS39
EMS42
N2, day 20
hsf-1(sy441)
EMS21
EMS31
EMS49
hsf-1, day 26
EMS30
EMS40
hsf-1, day 32
EMS32
EMS48
b
N2, 72 hrs, 20°C
hsf-1(sy441)
EMS4
EMS21
EMS27
EMS30
EMS31
EMS32
EMS39
EMS40
EMS42
EMS48
EMS49
c
N2, 60 hrs, 25°C
hsf-1(sy441)
EMS4
EMS21
EMS27
EMS30
EMS31
EMS32
EMS39
EMS40
EMS42
EMS48
EMS49

Supplementary Fig. 9 Phenotypic analysis of long-lived non-CID mutants.

(**a-c**) Long-lived non-CID mutant strains did not enter CID at 9 °C but exhibited developmental arrest along with *hsf-1(sy441)* mutants at 25 °C. Images were obtained at 60 h (**c**), 84 h (**b**), and on days 14, 20, 26 and 32, respectively (**a**). n ≥ 3 biological replicates with more than 50 animals (**a**–**c**). Scale bars, 1 mm. Arrows indicate arrested worms (**a**).

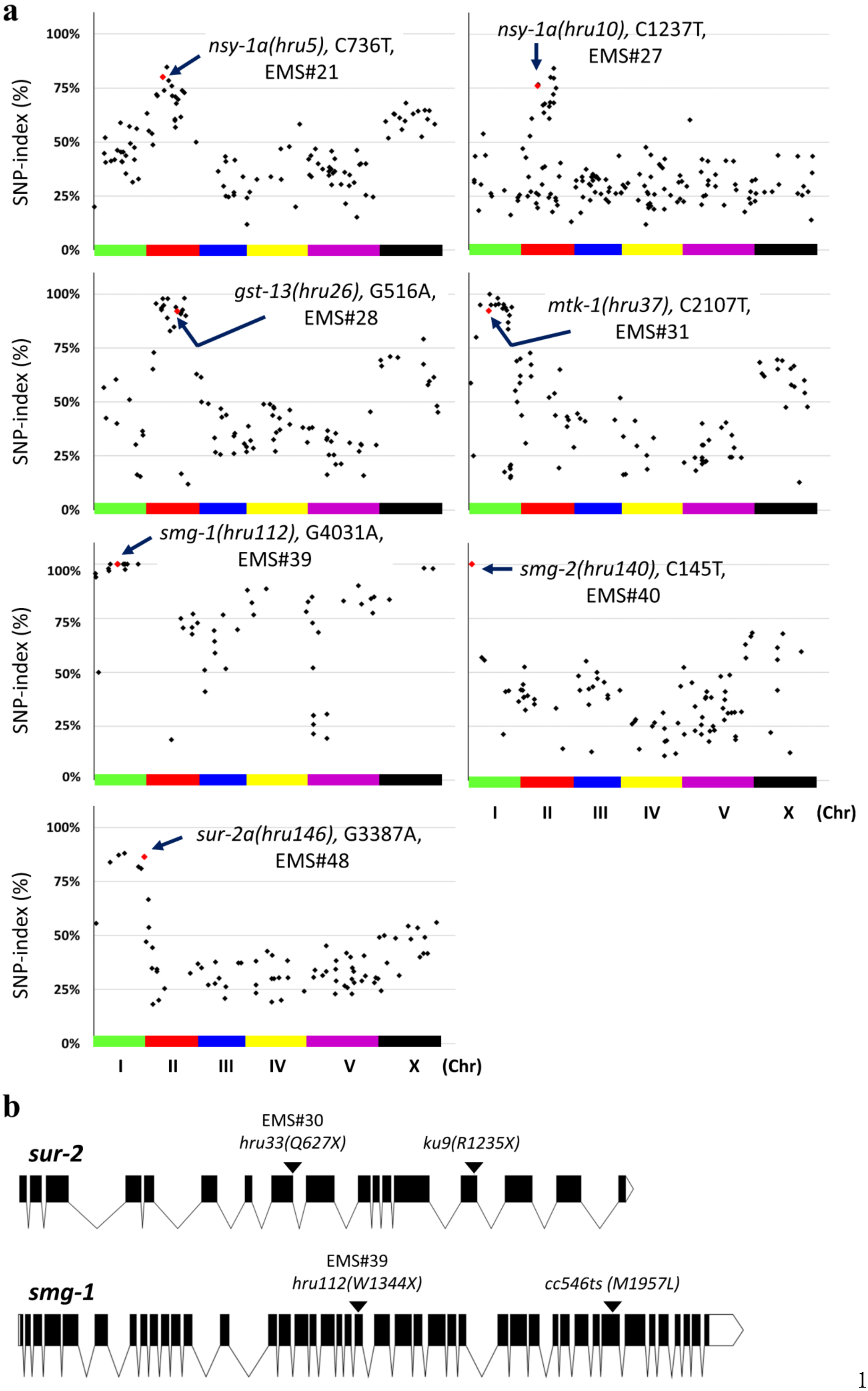
a
nsy-1a(hru5), C736T, EMS#21
nsy-1a(hru10), C1237T, EMS#27
gst-13(hru26), G516A, EMS#28
mtk-1(hru37), C2107T, EMS#31
smg-1(hru112), G4031A, EMS#39
smg-2(hru140), C145T, EMS#40
sur-2a(hru146), G3387A, EMS#48
SNP-index (%)
100%
75%
50%
25%
0%
I II III IV V X (Chr)
b
sur-2
EMS#30
hru33(Q627X)
ku9(R1235X)
smg-1
EMS#39
hru112(W1344X)
cc546ts (M1957L)

Supplementary Fig. 10 Candidates of regulators of *hsf-1(sy441)* mutant CID identified by MutMap analysis.

(a) SNP-index plots for each long-lived mutant. The red dots indicate the unique nonsense mutations in the quantitative trait loci (QTL) of each EMS mutant, whereas black dots indicate the identified non-synonymous single nucleotide polymorphisms (SNPs). The Y-axis represents the SNP index (SNP reads/total reads). Different colours on the X-axis correspond to each chromosome. (**b)** Schematic representations of mutations in *sur-2(hru33), sur-2(ku9), smg-1(hru112),* and *smg-1(cc546)*. Source data are provided as a Source Data file. The exon-intron boundaries were generated by Exon-Intron Graphic Maker version 4 (http://wormweb.org/exonintron, Bhatla, Nikhil. 2012).

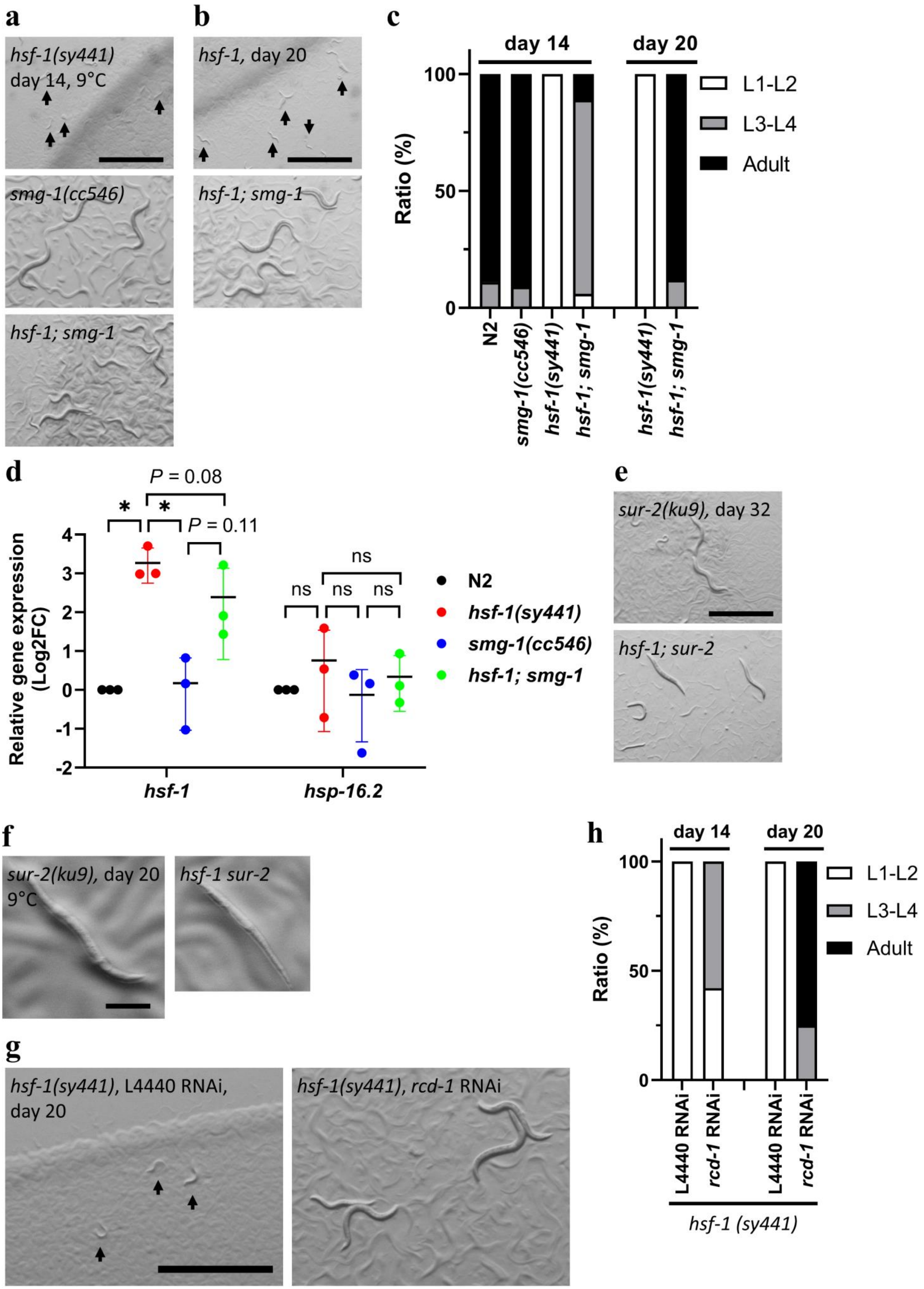
a
hsf-1(sy441)
day 14, 9°C
smg-1(cc546)
hsf-1; smg-1
b
hsf-1, day 20
hsf-1; smg-1
c
day 14
day 20
Ratio (%)
100
50
0
L1-L2
L3-L4
Adult
N2
smg-1(cc546)
hsf-1(sy441)
hsf-1; smg-1
hsf-1(sy441)
hsf-1; smg-1
d
P = 0.08
*
*
P = 0.11
ns
ns
ns
ns
Relative gene expression
(Log2FC)
4
3
2
1
0
-1
-2
hsf-1
hsp-16.2
N2
hsf-1(sy441)
smg-1(cc546)
hsf-1; smg-1
e
sur-2(ku9), day 32
hsf-1; sur-2
f
sur-2(ku9), day 20
9°C
hsf-1 sur-2
g
hsf-1(sy441), L4440 RNAi,
day 20
hsf-1(sy441), rcd-1 RNAi
h
day 14
day 20
Ratio (%)
100
50
0
L1-L2
L3-L4
Adult
L4440 RNAi
rcd-1 RNAi
L4440 RNAi
rcd-1 RNAi
hsf-1 (sy441)

Supplementary Fig. 11 Roles of CID regulator genes in the *hsf-1(sy441)* mutant.

(**a–c**) A mutation of *smg-1* prevented CID induction in *hsf-1*(*sy441*). **(d)** Relative gene expression of *hsf-1* and *hsp-16.2* was measured by qRT-PCR and normalised to N2. n = 3 biological replicates. Each dot represents one biological replicate. N2 (wild-type): black, *hsf-1(sy441)*: red, *smg-1(cc546)*: blue, *hsf-1;smg-1*: green. Bars indicate the mean, and error bars indicate the standard deviation (SD). Asterisk indicates $P < 0.001$ by 2-way ANOVA test. ns: Not significant. **(f)** *sur-2(ku9)* and *hsf-1; sur-2* mutants exhibited a rod-like lethal phenotype. **(g, h)** RNAi of *rcd-1/T24E12.5* inhibited CID induction in *hsf-1(sy441)* mutants. Images were obtained on days 14 (**a**), 20 (**b, f, g**), and 32 (**e**) at 9 °C. n ≥3 biological replicates with more than 50 animals (**a–c, e–h**). Graphs represent one of the biological replicates (**c, h**). White indicates the relative population of L1-L2 larvae, grey that of L3-L4 larvae, diagonal lines that of dauer and black that of adult worms. Scale bars, 0.1 mm (**f**) and 1 mm (**a, b, e, g**). Arrows indicate arrested worms (**a, b, g**). Source data are provided as a Source Data file.

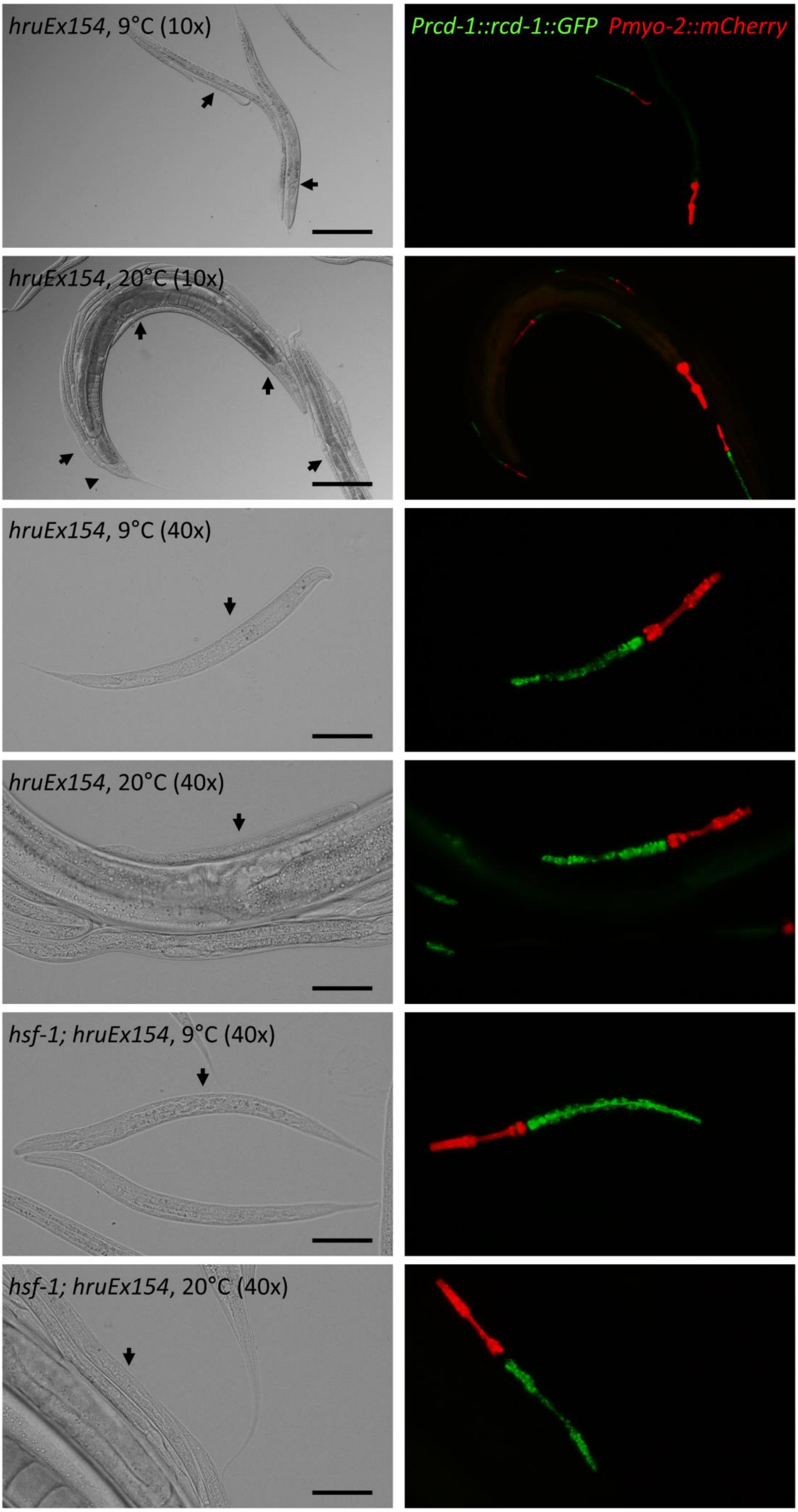
hruEx154, 9°C (10x)
Prcd-1::rcd-1::GFP Pmyo-2::mCherry
hruEx154, 20°C (10x)
hruEx154, 9°C (40x)
hruEx154, 20°C (40x)
hsf-1; hruEx154, 9°C (40x)
hsf-1; hruEx154, 20°C (40x)

Supplementary Fig. 12 The *rcd-1* gene is expressed in early larval stages.
RCD-1::GFP was expressed in the intestines of early larvae (L1 to L2) but not in later larval (L4) stages and adult worms. Expression levels and patterns of RCD-1::GFP were not altered by temperature or the *hsf-1* mutation. *Pmyo-2::mcherry* was used as a co-injection marker. *Prcd-1::rcd-1::gfp*: green, *Pmyo-2::mcherry*: red. Scale bar, 50 (magnitude 40 x) and 200 µm (magnitude 10 x). Arrows indicate *mcherry* (+) worms.

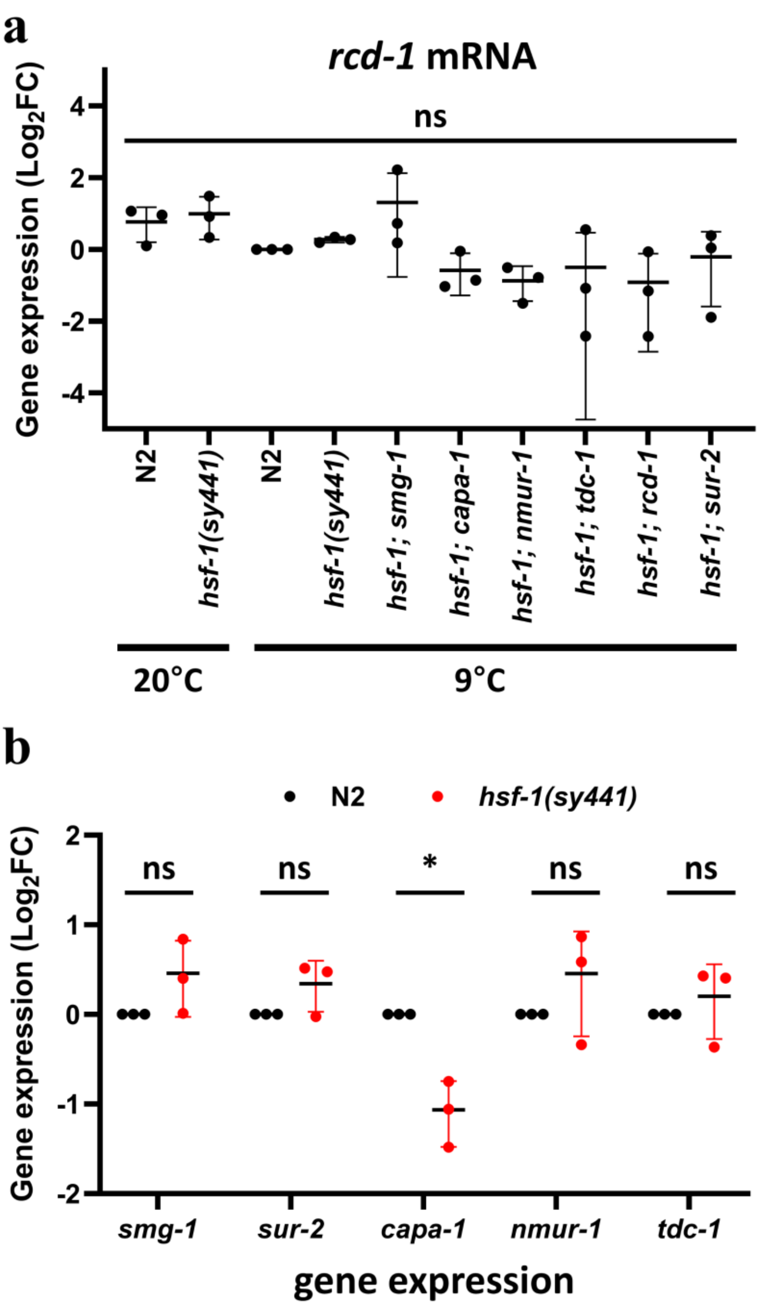

Supplementary Fig. 13 qRT-PCR analysis for regulator genes of *hsf-1(sy441)* mutant CID.

(a) Relative gene expression of *rcd-1* was measured by qRT-PCR and normalised to wild-type (N2) animals grown at 9 °C. **(b)** Relative gene expression of CID regulators was measured by qRT-PCR and normalised to wild-type (N2) animals grown at 9 °C. (**a, b**) n = 3 biological replicates. Each dot represents one biological replicate. Bars indicate the mean, and error bars indicate the standard deviation (SD). Black dots indicate N2 (wild-type) and red *hsf-1(sy441)*. Asterisk indicates $P < 0.001$ by multiple unpaired student *t*-test. ns: Not significant. Source data are provided as a Source Data file.

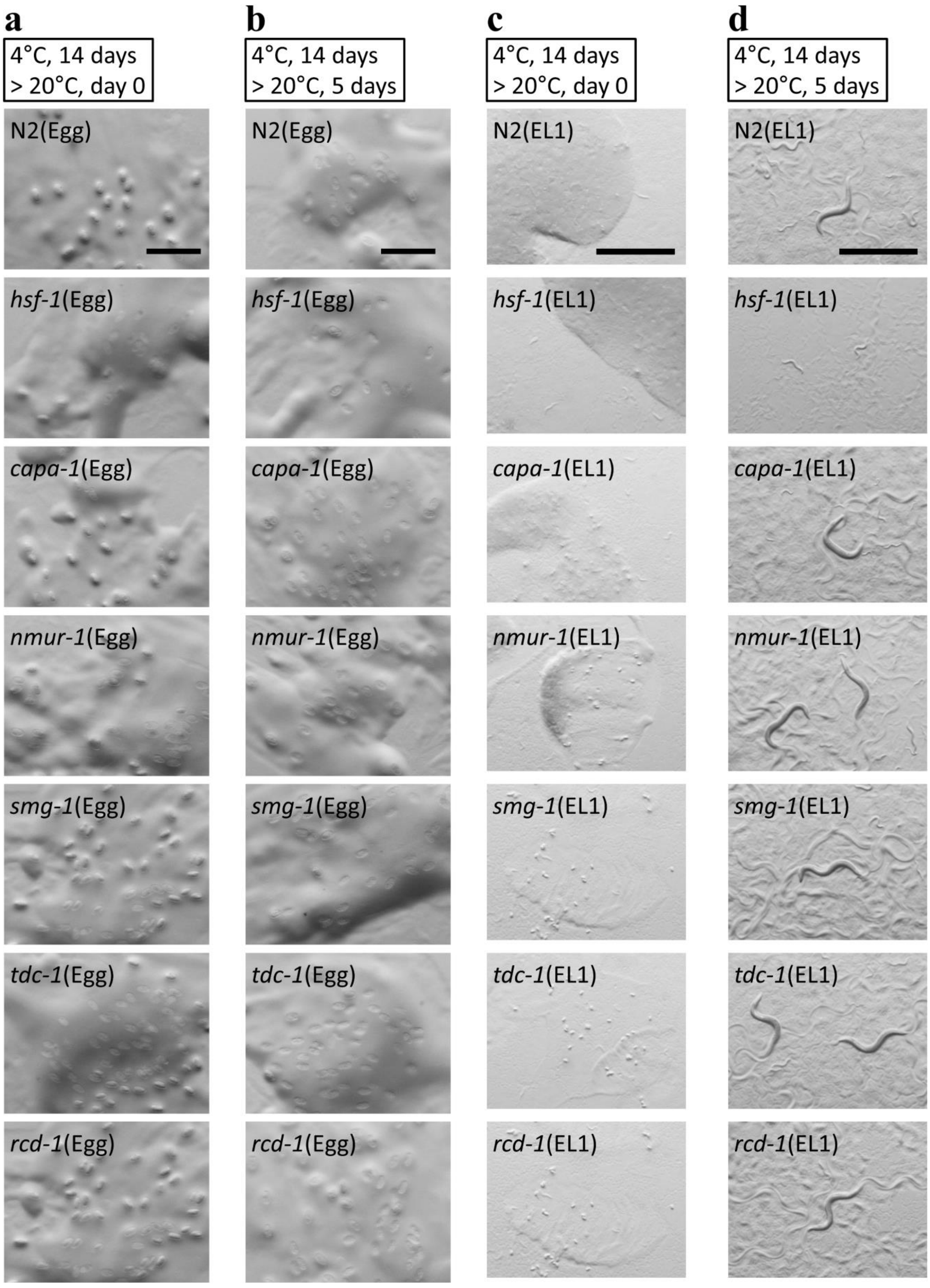

a
4°C, 14 days
> 20°C, day 0
b
4°C, 14 days
> 20°C, 5 days
c
4°C, 14 days
> 20°C, day 0
d
4°C, 14 days
> 20°C, 5 days
N2(Egg)
N2(Egg)
N2(EL1)
N2(EL1)
hsf-1(Egg)
hsf-1(Egg)
hsf-1(EL1)
hsf-1(EL1)
capa-1(Egg)
capa-1(Egg)
capa-1(EL1)
capa-1(EL1)
nmur-1(Egg)
nmur-1(Egg)
nmur-1(EL1)
nmur-1(EL1)
smg-1(Egg)
smg-1(Egg)
smg-1(EL1)
smg-1(EL1)
tdc-1(Egg)
tdc-1(Egg)
tdc-1(EL1)
tdc-1(EL1)
rcd-1(Egg)
rcd-1(Egg)
rcd-1(EL1)
rcd-1(EL1)

Supplementary Fig. 14 The *hsf-1(sy441)* mutant CID regulator genes were not involved in CID at 4 °C.

(**a, b**) Eggs of mutants of CID regulator genes were unhatched at 4 °C and not rescued by warming to 20 °C after cold exposure. (**c, d**) Cold treatment at 4 °C in early L1 timing induced diapause in mutants of CID regulator genes as well as wild-type animals. Scale bars, 200 μm (**a, b**) and 1 mm (**c, d**).

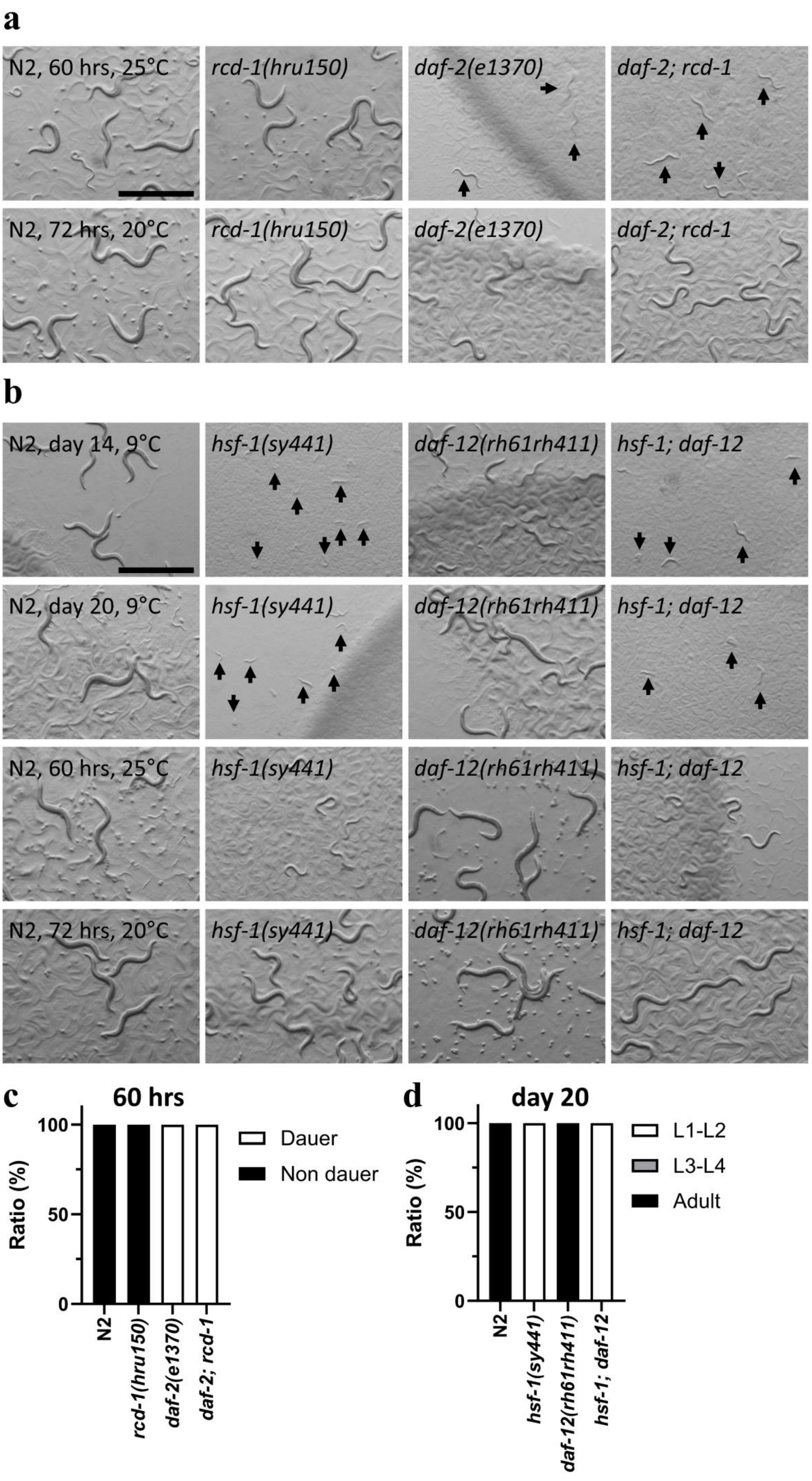
a
N2, 60 hrs, 25°C
rcd-1(hru150)
daf-2(e1370)
daf-2; rcd-1
N2, 72 hrs, 20°C
rcd-1(hru150)
daf-2(e1370)
daf-2; rcd-1
b
N2, day 14, 9°C
hsf-1(sy441)
daf-12(rh61rh411)
hsf-1; daf-12
N2, day 20, 9°C
hsf-1(sy441)
daf-12(rh61rh411)
hsf-1; daf-12
N2, 60 hrs, 25°C
hsf-1(sy441)
daf-12(rh61rh411)
hsf-1; daf-12
N2, 72 hrs, 20°C
hsf-1(sy441)
daf-12(rh61rh411)
hsf-1; daf-12
c
60 hrs
Ratio (%)
100
50
0
Dauer
Non dauer
N2
rcd-1(hru150)
daf-2(e1370)
daf-2; rcd-1
d
day 20
Ratio (%)
100
50
0
L1-L2
L3-L4
Adult
N2
hsf-1(sy441)
daf-12(rh61rh411)
hsf-1; daf-12

Supplementary Fig. 15 CID of the *hsf-1(sy441)* mutant and dauer regulatory mechanisms are paralle l. **(a, c)** The mutation *rcd-1(hru150)* did not inhibit dauer formation in *daf-2(e1370)* mutants at 25 °C. (**b, d**) The mutation *daf-12(rh61rh411)*, a master regulator of dauer formation, did not suppress CID i nduction in *hsf-1(sy441)* mutants at 9 °C. Images were obtained at 60 and 72 h (**a**), and on days 14 an d 20 (**b**).n ≥ 3 biological replicates with more than 50 animals. Graphs represent one of the biological replicates. White indicates the relative population of dauer larvae, and black that of non-dauer worms (**b**). White indicates the relative population of L1-L2 larvae, grey that of L3-L4 larvae, diagonal lines that of dauer and black that of adult worms (**d**). Scale bars, 1 mm (**a, b**). Arrows indicate dauer (**a**) an d arrested worms (**b**). Source data are provided as a Source Data file.

Supplementary Table 1. Developmental phenotypes of several mutants at 9 °C.

**Chaperone genes (mutant alelle)**	**Gene class**	**Phenotype at 9 °C**
N2		Adult
daf-41(ok3052)	Chaperone	Adult
hsf-1(sy441)	Chaperone	**Developmental arrest**
hsp-12.1(ok3622)	Chaperone	Adult
hsp-12.2(ok3638)	Chaperone	Adult
hsp-12.3(ok3095)	Chaperone	Adult
hsp-12.6(ok1077)	Chaperone	Adult
hsp-16.2(gk249)	Chaperone	Adult
hsp-16.48(ok577)	Chaperone	Adult
hsp-3(ok1083)	Chaperone	**Slower development**
hsp-4(gk514)	Chaperone	**Adult, Sterile**
hsp-43(ok647)	Chaperone	Adult
hsp-6(tm515)/unc-62(e644)	Chaperone	Adult
hsp-70(tm2318)	Chaperone	Adult
hsp-90(p673)	Chaperone	Adult

**Genes (mutant alelle)**	**Gene class**	**Phenotype at 9 °C**
paqr-1(tm3262)	Adiponectin receptor	Adult
paqr-2(tm3410)	Adiponectin receptor	**Slower development, Sterile**
paqr-3(ok2229)	Adiponectin receptor	Adult
hlh-30(tm1978)	Autophagy	Adult
daf-10(e1387)	Chemosensory	Adult
xdh-1(ok3234)	Cold acclimation	Adult
trpa-1(ok999)	Cold sensation	Adult
eat-2(ad465)	Dietary restrictions	Adult
atf-6(ok551)	ER stress response	Adult
ire-1(ok799)	ER stress response	**Slower development**
pek-1(ok275)	ER stress response	Adult
xbp-1(zc12)	ER stress response	Adult
daf-16(mgDf50)	Insulin signaling	Adult
daf-2(e1370)	Insulin signaling	Adult
pkc-1(ok563)	Protein Kinase C	Adult
pkc-2(ok328)	Protein Kinase C	Adult
daf-12(rh61rh411)	Steroidal	Adult
daf-5(e1386)	TGF-β	Adult

Supplementary Table 2. Developmental and behavioural phenotypes of EMS strains

EMS ID	Body shape	Locomotion	Reproduction
#30			egl, partially sterile
#31			egl, partially sterile
#32			egl
#36	dpy		
#37			egl, partially sterile
#39	pvu		
#42			partially sterile
#48	dpy		egl
#49		dwelling	

dpy: dumpy, pvu: protruding vulva, egl: egg-laying defective

Supplementary Table 3. Strains and chemicals used in this study

**Bacterial strains**	**Source**	**Identifier**
E. coli	Caenorhabditis Genetics Center (CDC)	WormBase: OP50

**Nematode strains**	**Source**	**Identifier**
N2	CGC	
atf-6(ok551)	CGC	AA1423
daf-10(e1387)	CGC	AA2674
daf-12(rh61rh411)	CGC	AA003
daf-16(mgDf50)	CGC	AA60
daf-2(e1370)	CGC	MZ230
daf-41(ok3052)	CGC	AA1780
daf-5(e1386)	CGC	AA4397
eat-2(ad465)	CGC	AA1409
hlh-30(tm1978)	CGC	AA3658
hsf-1(sy441)	CGC	MZ227
hsp-12.1(ok3622)	CGC	RB2600
hsp-12.2(ok3638)	CGC	RB2612
hsp-12.3(ok3095)	CGC	AA3985
hsp-12.6(ok1077)	CGC	RB1098
hsp-16.2(gk249)	CGC	VC475
hsp-16.48(ok577)	CGC	RB791
hsp-3(ok1083)	CGC	AA3627

hsp-4(gk514)	CGC	AA3628
hsp-43(ok647)	CGC	RB825
hsp-6(tm515)/unc-62(e644)	CGC	FX00515
hsp-70(tm2318)	CGC	FX02318
hsp-90(p673)	CGC	MZ228
ire-1(ok799)	CGC	MZ229
paqr-1(tm3262)	CGC	MZ258
paqr-2(tm3410)	CGC	MZ259
paqr-3(ok2229)	CGC	MZ260
pek-1(ok275)	CGC	AA1563
pkc-1(ok563)	CGC	AA2637
pkc-2(ok328)	CGC	AA2638
trpa-1(ok999)	CGC	AA3314
xbp-1(zc12)	CGC	MZ231
xdh-1(ok3234)	CGC	MZ470
drSi13	This paper	MZ598
drSi28	This paper	MZ599
hsf-1(sy441); drSi13	Morton et al. 2012	MZ600
hsf-1(sy441); drSi28	Morton et al. 2012	MZ601
hsf-1(sy441); hsp-90(p673)	This paper	MZ616
hsf-1(sy441); njEx394[ges-1p::hsf-1 cDNA (1 ng/ul), ges-1p::NLS::gfp)	Sugi et al. 2011	MZ420
hsf-1(sy441); njEx395[unc-14p::hsf-1 cDNA (1 ng/ul), ges-1p::NLS::gfp)	Sugi et al. 2011	MZ421
njEx394[ges-1p::hsf-1 cDNA (1 ng/ul), ges-1p::NLS::gfp)	This paper	MZ429
njEx395[unc-14p::hsf-1 cDNA (1 ng/ul), ges-1p::NLS::gfp)	This paper	MZ430
hsf-1(ljt3[hsf-1::degron::gfp]) I; ieSi57 II; unc-119(ed3) III.	Edwards et al. 2021	JTL611
unc-31(e928)	CGC	MZ264
unc-13(e51)	CGC	MZ263
hsf-1(sy441); unc-13(e51)	This paper	MZ290
hsf-1(sy441); unc-31(e928)	This paper	MZ291
hsf-1(sy441); daf-2(e1370)	This paper	MZ292
hsf-1(sy441); bas-1(ad446)	This paper	MZ443
hsf-1(sy441); cat-2(n4547)	This paper	MZ442
hsf-1(sy441); tbh-1(ok1196)	This paper	MZ444

hsf-1(sy441); tdc-1(ok914)	This paper	MZ445
hsf-1(sy441); tph-1(mg280)	This paper	MZ441
bas-1(ad446)	CGC	MZ455
cat-2(n4547)	CGC	MZ454
tbh-1(ok1196)	CGC	MZ456
tdc-1(ok914)	CGC	MZ457
tph-1(mg280)	CGC	MZ453
capa-1(ok3065)	CGC	MZ530
hsf-1(sy441); capa-1(ok3065)	This paper	MZ536
nmur-1(ok1387)	CGC	MZ469
hsf-1(sy441); nmur-1(ok1387)	This paper	MZ274
hsf-1(sy441); ldIs7 [skn-1b/c::GFP + rol-6(su1006)]	This paper	MZ271
hsf-1(sy441); muEx265[HSF-1p::HSF-1 cDNA + myo-3::GFP]	This paper	MZ285
hsf-1(sy441); muIs61 [(pKL78) daf16::GFP + rol-6(su1006)].	This paper	MZ317
hsf-1(sy441); sqIs19[hlh-30p::hlh-30::gfp+rol-6(su1006)]	This paper	MZ299
hsf-1(sy441); uthIs270[rab-3p::xbp-1s (constitutively active) + myo-2p::tdTomato]	This paper	MZ283
hsf-1(sy441); wuEx217[Pskn-1b::skn-1b::GFP; rol-6]	This paper	MZ281
daf-16(mu86); muIs61 [(pKL78) daf16::GFP + rol-6(su1006)].	CGC	MZ244
ldIs7 [skn-1b/c::GFP + rol-6(su1006)]	An et al. 2003	MZ261
muEx265[HSF-1p::HSF-1 cDNA + myo-3::GFP]	CGC	MZ243
sqIs19[hlh-30p::hlh-30::GFP + rol-6(su1006)]	CGC	MZ242
uthIs270[rab-3p::xbp-1s + myo-2p::tdTomato]	CGC	MZ245
wuEx217[Pskn-1b::skn-1b::GFP; rol-6]	Keith Blackwell's lab	MZ265
rcd-1(hru150)	This paper	MZ459
hsf-1(sy441); rcd-1(hru150)	This paper	MZ464
sur-2(ku9)	CGC	MZ509
smg-1(cc546)	CGC	MZ510

hsf-1(sy441) sur-2(ku9)	This paper	MZ511
hsf-1(sy441); smg-1(cc546)	This paper	MZ513
rcd-1(hru150); daf-2(e1370)	This paper	MZ611
hsf-1(sy441); daf-12(rh61rh411)	This paper	MZ610
hsf-1(sy441)I; hruEx154 [Prcd-1::rcd-1::GFP::unc-54 3'UTR, pCFJ90] lot#01	This paper	MZ524
hsf-1(sy441)I; hruEx155 [Prcd-1::rcd-1::GFP::unc-54 3'UTR, pCFJ90] lot#02	This paper	MZ525
hsf-1(sy441)I; hruEx156 [Prcd-1::rcd-1::GFP::unc-54 3'UTR, pCFJ90] lot#03	This paper	MZ526
hruEx154 [Prcd-1::rcd-1::GFP::unc-54 3'UTR, pCFJ90] lot#01	This paper	MZ515
hruEx155 [Prcd-1::rcd-1::GFP::unc-54 3'UTR, pCFJ90] lot#02	This paper	MZ516
hruEx156 [Prcd-1::rcd-1::GFP::unc-54 3'UTR, pCFJ90] lot#03	This paper	MZ517
hsf-1(sy441) EMS1-1	This paper	MZ468
hsf-1(sy441) EMS1-2	This paper	MZ469
hsf-1(sy441) EMS1-3	This paper	MZ470
hsf-1(sy441) EMS1-4	This paper	MZ471
hsf-1(sy441) EMS1-5	This paper	MZ472
hsf-1(sy441) EMS1-6	This paper	MZ473
hsf-1(sy441) EMS1-7	This paper	MZ474
hsf-1(sy441) EMS1-8	This paper	MZ475
hsf-1(sy441) EMS1-9	This paper	MZ476
hsf-1(sy441) EMS1-10	This paper	MZ477
hsf-1(sy441) EMS1-11	This paper	MZ478
hsf-1(sy441) EMS1-12	This paper	MZ479
hsf-1(sy441) EMS1-13	This paper	MZ480
hsf-1(sy441) EMS1-14	This paper	MZ481
hsf-1(sy441) EMS1-15	This paper	MZ482
hsf-1(sy441) EMS1-16	This paper	MZ483
hsf-1(sy441) EMS1-17	This paper	MZ484
hsf-1(sy441) EMS1-18	This paper	MZ485
hsf-1(sy441) EMS1-19	This paper	MZ486
hsf-1(sy441) EMS1-20	This paper	MZ487
hsf-1(sy441) EMS1-21	This paper	MZ488
hsf-1(sy441) EMS1-22	This paper	MZ489

hsf-1(sy441) EMS1-23	This paper	MZ490
hsf-1(sy441) EMS1-24	This paper	MZ491
hsf-1(sy441) EMS1-25	This paper	MZ492
hsf-1(sy441) EMS1-26	This paper	MZ493
hsf-1(sy441) EMS1-27	This paper	MZ494
hsf-1(sy441) EMS1-28	This paper	MZ495
hsf-1(sy441) EMS1-29	This paper	MZ496
hsf-1(sy441) EMS1-30	This paper	MZ497
hsf-1(sy441) EMS1-31	This paper	MZ498
hsf-1(sy441) EMS1-32	This paper	MZ499
hsf-1(sy441) EMS1-33	This paper	MZ500
hsf-1(sy441) EMS1-34	This paper	MZ501
hsf-1(sy441) EMS1-35	This paper	MZ502
hsf-1(sy441) EMS1-36	This paper	MZ503
hsf-1(sy441) EMS1-37	This paper	MZ504
hsf-1(sy441) EMS1-38	This paper	MZ505
hsf-1(sy441) EMS1-39	This paper	MZ506
hsf-1(sy441) EMS1-40	This paper	MZ507
hsf-1(sy441) EMS1-41	This paper	MZ508
hsf-1(sy441) EMS1-42	This paper	MZ509
hsf-1(sy441) EMS1-43	This paper	MZ510
hsf-1(sy441) EMS1-44	This paper	MZ511
hsf-1(sy441) EMS1-45	This paper	MZ512
hsf-1(sy441) EMS1-46	This paper	MZ513
hsf-1(sy441) EMS1-47	This paper	MZ514
hsf-1(sy441) EMS1-48	This paper	MZ515
hsf-1(sy441) EMS1-49	This paper	MZ516
hsf-1(sy441) EMS1-4, 4x Outcrossed	This paper	MZ476
hsf-1(sy441) EMS1-21, 4x Outcrossed	This paper	MZ478
hsf-1(sy441) EMS1-27, 4x Outcrossed	This paper	MZ479
hsf-1(sy441) EMS1-30, 4x Outcrossed	This paper	MZ481
hsf-1(sy441) EMS1-31, 4x Outcrossed	This paper	MZ482
hsf-1(sy441) EMS1-32, 4x Outcrossed	This paper	MZ483
hsf-1(sy441) EMS1-39, 4x Outcrossed	This paper	MZ485
hsf-1(sy441) EMS1-40, 4x Outcrossed	This paper	MZ486
hsf-1(sy441) EMS1-42, 4x Outcrossed	This paper	MZ487
hsf-1(sy441) EMS1-48, 4x Outcrossed	This paper	MZ488
hsf-1(sy441) EMS1-49, 4x Outcrossed	This paper	MZ489

Chemicals	Source	Identifier
Sodium hydroxide	Kanto Chemical	37184-00
Proteinase K solution	Kanto Chemical	34060-97
Cholesterol	Kanto Chemical	07331-00
Potassium phosphate dibasic	Merck	24-5240
Potassium phosphate monobasic	Merck	24-5260
Magnesium sulfate	Merck	19-0480
Disodium Hydrogenphosphate	Merck	28-3720
Calcium chloride	Merck	05-0590
Agar, powder	Nacalai Tesque	01028-14
Sodium Chloride	Nacalai Tesque	31-320-76
dNTPs Mixture	NIPPON GENE	312-07271
Bacto Tryptone	Thermo Fisher Scientific	211705
Yeast Extract	Thermo Fisher Scientific	211929
Bacto Peptone	Thermo Fisher Scientific	211677
2'-Deoxy-5-fluorouridine (FUDR)	Tokyo Chemical Industry Co., Ltd.	D2235
indole-3-acetic acid	Merck	I3750

Critical commercial assays	Source	Identifier
ISOSPIN Tissue DNA	NIPPON GENE	316-08891
Phusion High-Fidelity DNA Polymerase	New England Biolabs	M0530
NucleoSpin® RNA Plus	Takara Bio	U0984B
SuperScript™ III Reverse Transcriptase	Thermo Fisher Scientific	18080044
PowerTrack SYBR Master Mix	Thermo Fisher Scientific	A46109

Supplementary Table 4. Primers for genotyping and qRT-PCR

Primer Code for Genotyping	Sequence (5'-3')	Comments
bas-1(ad446) Fw	TTACAATTAGGCCGCAAACC	
bas-1(ad446) Rv	CTGCCTACTTGCCTGCCTAC	
capa-1(ok3065) Fw	CTAAAAACGCATGCCTGGAT	
capa-1(ok3065) Rv	ACGGAACAGATCTCCTCGAA	
cat-2(n4547) Fw	ACTTCCGTCCGTCTTGAGAA	
cat-2(n4547) Rv	CGACGTGGATTTTTGGAGTT	
daf-12(rh61) Fw	CCAACAATTAAGGGGCAA	
daf-12(rh61) Rv	CCAACAATTAAGGGGCA	
daf-12(rh61) SNP site1	TGACGGAAACGTTTTACC	For mismatch amplification

daf-12(rh61) SNP site2	TTGACGGAAACGTTTTAC	For mismatch amplification
hsp-90(p673) BmrI Fw	AGGAGAAGAAGGAGGGAGAG	WT fragment can be digested by BmrI (approximate length of the products are 200 and 400 bp)
hsp-90(p673) BmrI Rv	GTAGCGAAGGAAATCGGAAAG	
nmur-1(ok1387) Fw	ATGTAGCGGTGTGTCATCCA	
nmur-1(ok1387) Rv	GAAGAAAGCCGAAACGATTG	
smg-1(hru112) BglI Fw	GCACATGAGTCGAGCCAGTA	MT fragment can be digested by BglI (approximate length of the products are 250 and 300 bp)
smg-1(hru112) BglI Rv	TATTGGGTGCCAGAATGTTG	
sur-2(hru33) Fw	GACGTTCCGATCGAAGAAAA	
sur-2(hru33) Rv	TAGGCATGTAGGTGGGGAAG	
sur-2(hru33) SNPsite1	CTTTTTTTCAAAATCTAACCTAATG	For mismatch amplification
sur-2(hru33) SNPsite2	CATCTGGTGAACATCAGG	For mismatch amplification
T24E12.5/rcd-1(hru150) Fw	TCCTTGGAAATGCTGTGTCA	
T24E12.5/rcd-1(hru150) Rv	TTTGGAATGATACCCCAACAA	
T24E12.5/rcd-1(hru150) SNP site1	TCTTTTTCGGGTTCATATCA	For mismatch amplification
T24E12.5/rcd-1(hru150) SNP site2	GTCTTTTTCGGGTTCATATC	For mismatch amplification
tbh-1(ok1196) Fw	GAACGCCAGTTGGTTGATTT	
tbh-1(ok1196) Rv	CATGTCATTGATGGCTGGAC	
tdc-1(ok914) Fw	AAATGGTTTACGGGCTTGG	
tdc-1(ok914) Rv	ATGGTTGGCCATGTTGAGAT	
tph-1(mg280) Fw	CCCCTCTCAACCTCATTTCA	
tph-1(mg280) Rv	GCGAACGTATTGAGTGCAGA	

**Primer Code for qRT-PCR**	**Sequence (5'-3')**	**Comments**
ama-1 Fw	GGATGGAATGTGGGTTGAGA	Internal control
ama-1 Rv	CGGATTCTTGAATTTCGCGC	Internal control
hsf-1 Fw	GCATAACAATATGAATAGCATGGTC	Target gene
hsf-1 Rv	GACGTCCTTGTACAAAACACGGATG	Target gene
hsp-16.2 Fw	ACTTTACCACTATTTCCGTCCAGC	Target gene

hsp-16.2 Rv	CCTTGAACCGCTTCTTTCTTT	Target gene
sur-2 Fw	CGGAGACTTTTTGAGGAATACG	Target gene
sur-2 Rv	AGAGGTTGGTGAGCTTTGACAT	Target gene
smg-1 Fw	ACATGGCGATGTTCTCATTG	Target gene
smg-1 Rv	CCGTCTGTAGAAGATGATGCAG	Target gene
rcd-1 Fw	AGAAAGCACGTACTTCGGAATC	Target gene
rcd-1 Rv	GGGTGTTACATTCTCCTCTTGG	Target gene
capa-1 Fw	CCATCATCTGCTCTCTTAGTGC	Target gene
capa-1 Rv	TTCCCAATACGTGGAGTGTAGA	Target gene
nmur-1 Fw	TCTTTGCACTCCCACTGTTTAC	Target gene
nmur-1 Rv	AAACGGAAGGTAGCAGATGAAG	Target gene
tdc-1 Fw	GAGCTATGGCTTCATGTTGATG	Target gene
tdc-1 Rv	ACGATCACGTACCCACATTGTA	Target gene

Supplementary methods

Cold-inducible diapause experiment

Worms were allowed to lay eggs on NGM plates, and the hatched animals were washed with M9 buffer. Approximately 100 eggs were transferred to OP50 bacterial lawns on fresh NGM plates and cultivated at 4 °C and 9 °C. Eggs were collected using the standard bleach treatment when the experiment involves the *sur-2(ku9)* mutant. The CID phenotypes of the strains were observed, and their population was classified based on body length, and egg carrying at 14, 20, 26, and 32 days, respectively. After 14 days of incubation at 4°C and 20, 40 and 60 days of incubation at 9°C, recovery from CID was observed at 20°C. Images were obtained using the binocular SZX10 (OLYMPUS) with a DP27 camera (OLYMPUS). We used ImageJ software to measure the body length of the worms.

Morphological analysis of CID

C. elegans embryos and larvae were mounted on 4% agar pads. Anaesthesia was not applied in order to preserve cellular integrity and facilitate cellular identification. Differential interference contrast (DIC) images were obtained using an Axio Imager M2 equipped with a Plan-Apochromat 63x/1.40 Oil DIC and Axiocam 506 mono digital camera and processed using Zen (Carl Zeiss) and Photoshop (Adobe) software.

Plasmid construction and transgenesis

To generate the *Prcd-1::rdc-1::gfp* fusion construct, an approximate 1-kb endogenous promoter and 3.4-kb genome region of the *rcd-1* gene was cloned into the pPD95.77 vector, which contained a green fluorescent protein (GFP) tag prior to *unc-54 3'UTR*. The whole sequence of the *Prcd-1::rdc-1::gfp::unc-54 3'UTR* plasmid is displayed below, with codons in capitals indicating coding sequences. Microinjection of the plasmid was performed with the co-injection marker pCFJ90 (*Pmyo-2::mCherry*) vector to generate *hruEx154* (*Prcd-1::rdc-1::gfp, Pmyo-2::mCherry).* The plasmid and strain were generated by SunyBiotech services (Fujian, China). The generated strain was outcrossed with N2 (wild-type) animals approximately four times before use.

Prcd-1::rcd-1::gfp

aagctagaattcaaggtttctattgactagtctcactagaaatacagtaatctaacagtattcctacatcaccatacaactccggttttaaaagtttaatat
ttcgaaatatttgttgatatcagaaacattttaaaaatcttcctgagaaaatataagagtgaggaagtgacatcaattccaaagatcgacatcataactg
ataacgagtcacaataaaacgcaacaattttcattttaatacgctcactttcctttcccgtttcagtaaccgcgaaatatttctgaaccactgaaaattgc
atattttcaatttttaagcttttgaaaataggttttgaagatttaaaaaaatcagcgggagtcttctcattttcaagaaaagttcaaattgaaaactgttaaa
aaaatcttaactgttggtgaaacagtttgtttatacctgatgcaatgcccggctttctccctcttttcttttttacaccaagaaaaatcgtttttttctcattttt
tttctttttgtctttttctttgttgatgtttttatctgatatatctgatacaaaatgtgtttttcatgtttctttttatgacttccttggaaatgctgtgtcaactttga
taactataattattgaagttgataaaacaaattgaaaaaataccaaactgaaatttggctgaaataaaacagcgaatttcaattaaaaaaaaaaaaacaa
aaaattcaacttttcacgctattaaaaatattggaacttgttttgctttgtttttagttttaaaatccacaccatgtaaatatgtagaggctgaaaattgagta
aagtgattttcagtgggaataacatccagcatttttttccggaaaacgcggagaggtcatcaagcgcactctaataaatatactcatttcaggagagc

ctgaataacgaATGGCTACATGTGTTTTAACTATAGCTCCCCCTGTGATTGAAGCTGTTGCTGCAT
CAGCCGTTGGACTTTCCGCTGCATCTGCCACAGGAGCTGGCGTTGCATCTGCCGCTGGAG
TTGTTGCTGCATCTGCCGCTGGAACTGGCGCTGCATCTACCGCTGGAGTTGTTGCTGCATC
TGCCGCTGGAGTTGGGGCTGGAGCTGTTGGGCTTACTCTCGTGCTCCCAGCTGTTTTGAT
GGGCGTGGGAGCTGGTTTGATAGTAGGTTCAATTGGTCTTTTAGTTTTGGGCCCACCTAA
AAGGAGAACATCAAACAATCAAACAGCGAGGCGCACTCCATGTAGCTCCACAAATTCTC
CTCGAAAATCTGAATCATGTTGGTATGAACCCGAAAAAGACCATTTCTACGTTTCCCAAA
AAGCACGTAATTCGGAATCAATTCGACTAACCGCCAATGTCATTCCCGGAGAAACATTGA
AAACATATGCTGAACTCAATGAAATACTgtgagttgttgttgtgacttcgttccaaaaatgattactatttgtgaatccccagt
gtggttctgaaacaattgcatatacatttcaccgatcgacacaaaatgattaaacgatgattttcatttttaagATTTAATCAACTTAATG
AAAATCGGACTGGATCCCCTGTGTTTCGAAAATCTGACTCTTATTGGTATGAACCTGAAA
AAGACCATTTCTACGTTTCCAAAGAAGCACGTAATTCGGAATCAATTCGACTAACCGCCA
ATGTCATTCCCGGAGAAACATTGAAAACATATGCTGAACTCAATGAAATACTgtgagttgttgttgt
gacttcgttccaaaaatgattactatttgtgaatccccagtgtggttctgaaacaattgcatatacatttcaccgatcgacacaaaatgattaaacgatg
attttcatttttaagATTTAATCAACTTAATGAAAATCGGACTGGATCCCCTGTGTTTCGAAAATCTG
ACTCTTATTGGTATGAACCTGAAAAAGACCATTTCTACGTTTCCAAAGAAGCACGTAATTC
GGAATCAATTCGACTAACCGCCAATGTCATTCCCGGAGAAACATTGAAAACATATGCTGA
ACTCAATGAAATACTgtgagttgttgttgtgacttcgttccaaaaatgattactatttgtgaatccccagtgtggttctgaaacaattgcat
atacatttcaccgatcgacacaaaatgattaaacgatgattttcatttttaagATTTAATCAACTTAATGAAAATCGGACTG
GATCCCCTGTGCTTCGAATATTTGACTCTTATTGGTATGAACCTGAAAAAGACCATTTCTA
CGTTTCCAAAAAAGCACGTACTTCGGAATCAATTCGACTAACCGCCAATGTCATTCCCGG
AGAAACATTGAAAACATATGCTGAACTCAATGAAATACTgtgagttgttgttgtgacttcgttccaaaaatgatta
ctatttgtgaatccccagtgtggttctgaaacaattgcatatacatttcaccgattgacataaaatgattaaacgatgtttttcatttttcagATTTAAT
AAACTCAATGAAAATCGGACTGGATCCCCTGTGCTTCGAATATCTGACTCTTATTGGTATG
AACCTGAAAAAGACCATTTCTACGTTTCCAAGAAAGCACGTACTTCGGAATCAATTCGAC
TAACCGCCAATGTCGTTTCCGGAGAAAAATGGAAAACATTTGCTGAACTCAAGGAATTAC
TAGGAAAgtaagttgttggggtatcattccaaaaagtgtcactgtttgcgattatgaaatatttatatttgattgaaatataataattgacgtaaaat
gataaaacgttgtctttcattcacaaagtcttaactgctgcaatttcccagaaaaataatgtttcgaatatgttttcagagtaaaacagtgttttcttcaaat
taatcactttcctcctttttctctatacctatgaaattactgttttcaacaggaaccaagacatgaatttggttttaatgaattctgaaattgtgttttgagttt
gaaatttgaaaaagcggttaaaaataattcaggcaaattacttttttttactaattccaatacttaagaacggatttgcaataaatacagaaaaggaaa
ataagtgcctaaaactactttaaaagtgtatcacgatgttttgatgtacgaatttaaacctaaaatctaaacctcaaagttgattgcactacgtgtcttaat
aaaatcttaaaaaaacatttttatagCCAACTTCGTGACGCTAACCAATCGAAACAAGATTCCTATCCAAG
AGGAGAATGTAACACCCCAGAGAAAAGAAATGAAAAAAACAATAATTTCGAGAGACAA
CAACCACGAAAATCGTTGCAAGATCAGCATTGGAATGTTCCTTTTGAATTCGAGAGAGAC
CCGAACAACCAAACAGGCCGAGAAAGAGAAGATTCATCCACTGACCAGTTTTGGATGAG
CGTTACAACTCCAAAAAACTTTACATACACCgtaagcttctttcaaaaatatagtcaccggagggttcaagagcttattt
aagaattgacaggtaagcgcgctagcccaacttgagcaaaggcttgcagcactaatagagaatatacggtaactaaccgacaatgtttggacgtc
agtttatttgcaaaaatcaaaaatgtttcgtacatacatataaaccattgttttatgaaaaaaagttttcagaaattacaagattgttttgagcattatccatg
aaaattcattatgtttgaatctagggatataacgaacgtaaagtttgcagATGGTAGGTCTTGCCAACAATGGACGCGAA

GGTTTCCTGCATTCGTTAATCAGACACGGAAGAGAGTTCACGGGCTTTCCATCGAATCGT
TTTCACAGAGATATTCTGGATGTAGTTCCACCATCCATACGCCAATTGATAAAAGAAATTC
GGAATTACAGCAACAGAGACAGGAGTAATGAGATAGAAAAAGAGTTACTTTCGGATAGG
TCATATCCTGgtaaacacatgtagttagaccgaactaatttttaatatttccagCTACACTGCAACTCCAGAAAAAGT
TTCGAGATTACATCCAGAAAATTGTAAACGATGAGAGCAACTTTTTGTACAGACGCCCTA
ATACAAACGGACAAACTCAAGTATTTTGGAAAATAAACAACGAGAAGGTTCTTGTGATTT
GTTTATCAGAAGACCCATATTTTCCTGAAGCTGGCAGGGATTCTGTTTCACCGGAAAGAA
ATATCAGAAAAATTATTACTATGTTTGTGAAAAGCAGGATAAGTGTGGACTCGATGATAAG
AGCAGACTCTCCGTTCAATACCCATGGTCgatccccgggattggccaaaggacccaaaggtatgtttcgaatgatacta
acataacatagaacattttcaggaggacccttgagggtaccggtagaaaaaATGAGTAAAGGAGAAGAACTTTTCACT
GGAGTTGTCCCAATTCTTGTTGAATTAGATGGTGATGTTAATGGGCACAAATTTTCTGTCA
GTGGAGAGGGTGAAGGTGATGCAACATACGGAAAACTTACCCTTAAATTTATTTGCACTA
CTGGAAAACTACCTGTTCCATGGGTAAGTTTAAACATATATATACTAACTAACCCTGATTAT
TTAAATTTTCAGCCAACACTTGTCACTACTTTCTGTTATGGTGTTCAATGCTTCTCGAGATA
CCCAGATCATATGAAACGGCATGACTTTTTCAAGAGTGCCATGCCCGAAGGTTATGTACA
GGAAAGAACTATATTTTTCAAAGATGACGGGAACTACAAGACACGTAAGTTTAAACAGTT
CGGTACTAACTAACCATACATATTTAAATTTTCAGGTGCTGAAGTCAAGTTTGAAGGTGAT
ACCCTTGTTAATAGAATCGAGTTAAAAGGTATTGATTTTAAAGAAGATGGAAACATTCTTG
GACACAAATTGGAATACAACTATAACTCACACAATGTATACATCATGGCAGACAAACAAA
AGAATGGAATCAAAGTTGTAAGTTTAAACATGATTTTACTAACTAACTAATCTGATTTAAA
TTTTCAGAACTTCAAAATTAGACACAACATTGAAGATGGAAGCGTTCAACTAGCAGACCA
TTATCAACAAAATACTCCAATTGGCGATGGCCCTGTCCTTTTACCAGACAACCATTACCTG
TCCACACAATCTGCCCTTTCGAAAGATCCCAACGAAAAGAGAGACCACATGGTCCTTCTT
GAGTTTGTAACAGCTGCTGGGATTACACATGGCATGGATGAACTATACAAATAGcattcgtagaa
ttccaactgagcgccggtcgctaccattaccaacttgtctggtgtcaaaaataataggggccgctgtcatcagagtaagtttaaactgagttctacta
actaacgagtaatatttaaattttcagcatctcgcgcccgtgcctctgacttctaagtccaattactcttcaacatccctacatgctctttctccctgtgctc
ccaccccctatttttgttattatcaaaaaacttctcttaatttctttgtttttagcttcttttaagtcacctctaacaatgaaattgtgtagattcaaaaatagaa
ttaattcgtaataaaaagtcgaaaaaaattgtgctccctccccccattaataataattctatcccaaaatctacacaatgttctgtgtacacttcttatgtttt
ttacttctgataaatttttttgaaacatcatagaaaaaaccgcacacaaaataccttatcatatgttacgtttcagtttatgaccgcaatttttatttcttcgca
cgtctgggcctctcatgacgtcaaatcatgctcatcgtgaaaaagttttggagtatttttggaatttttcaatcaagtgaaagtttatgaaattaattttcct
gcttttgctttttggggtttcccctattgtttgtcaagatttcgaggacggcgtttttcttgctaaaatcacaagtattgatgagcacgatgcaagaaagat
cggaagaaggtttgggtttgaggctcagtggaaggtgagtagaagttgataatttgaaagtggagtagtgtctatggggtttttgccttaaatgacag
aatacattcccaatataccaaacataactgtttcctactagtcggccgtacgggccctttcgtctcgcgcgtttcggtgatgacggtgaaaacctctga
cacatgcagctcccggagacggtcacagcttgtctgtaagcggatgccgggagcagacaagcccgtcagggcgcgtcagcgggtgttggcgg
gtgtcggggctggcttaactatgcggcatcagagcagattgtactgagagtgcaccatatgcggtgtgaaataccgcacagatgcgtaaggagaa
aataccgcatcaggcggccttaagggcctcgtgatacgcctatttttataggttaatgtcatgataataatggtttcttagacgtcaggtggcacttttc
ggggaaatgtgcgcggaacccctatttgtttatttttctaaatacattcaaatatgtatccgctcatgagacaataaccctgataaatgcttcaataatatt
gaaaaaggaagagtatgagtattcaacatttccgtgtcgcccttattcccttttttgcggcattttgccttcctgtttttgctcacccagaaacgctggtg
aaagtaaaagatgctgaagatcagttgggtgcacgagtgggttacatcgaactggatctcaacagcggtaagatccttgagagttttcgccccgaa

gaacgttttccaatgatgagcacttttaaagttctgctatgtggcgcggtattatcccgtattgacgccgggcaagagcaactcggtcgccgcataca
ctattctcagaatgacttggttgagtactcaccagtcacagaaaagcatcttacggatggcatgacagtaagagaattatgcagtgctgccataacc
atgagtgataacactgcggccaacttacttctgacaacgatcggaggaccgaaggagctaaccgcttttttgcacaacatgggggatcatgtaact
cgccttgatcgttgggaaccggagctgaatgaagccataccaaacgacgagcgtgacaccacgatgcctgtagcaatggcaacaacgttgcgc
aaactattaactggcgaactacttactctagcttcccggcaacaattaatagactggatggaggcggataaagttgcaggaccacttctgcgctcgg
cccttccggctggctggtttattgctgataaatctggagccggtgagcgtgggtctcgcggtatcattgcagcactggggccagatggtaagccct
cccgtatcgtagttatctacacgacggggagtcaggcaactatggatgaacgaaatagacagatcgctgagataggtgcctcactgattaagcatt
ggtaactgtcagaccaagtttactcatatatactttagattgatttaaaacttcatttttaatttaaaaggatctaggtgaagatcctttttgataatctcatg
accaaaatcccttaacgtgagttttcgttccactgagcgtcagaccccgtagaaaagatcaaaggatcttcttgagatcctttttttctgcgcgtaatct
gctgcttgcaaacaaaaaaaccaccgctaccagcggtggtttgtttgccggatcaagagctaccaactctttttccgaaggtaactggcttcagcag
agcgcagataccaaatactgtccttctagtgtagccgtagttaggccaccacttcaagaactctgtagcaccgcctacatacctcgctctgctaatcc
tgttaccagtggctgctgccagtggcgataagtcgtgtcttaccgggttggactcaagacgatagttaccggataaggcgcagcggtcgggctga
acggggggttcgtgcacacagcccagcttggagcgaacgacctacaccgaactgagatacctacagcgtgagcattgagaaagcgccacgctt
cccgaagggagaaaggcggacaggtatccggtaagcggcagggtcggaacaggagagcgcacgagggagcttccagggggaaacgcctg
gtatctttatagtcctgtcgggtttcgccacctctgacttgagcgtcgatttttgtgatgctcgtcaggggggcggagcctatggaaaaacgccagca
acgcggcctttttacggttcctggccttttgctggccttttgctcacatgttctttcctgcgttatcccctgattctgtggataaccgtattaccgcctttga
gtgagctgataccgctcgccgcagccgaacgaccgagcgcagcgagtcagtgagcgaggaagcggaagagcgcccaatacgcaaaccgcc
tctccccgcgcgttggccgattcattaatgcagctggcacgacaggtttcccgactggaaagcgggcagtgagcgcaacgcaattaatgtgagtt
agctcactcattaggcaccccaggctttacactttatgcttccggctcgtatgttgtgtggaattgtgagcggataacaatttcacacaggaaacagct
atgaccatgattacgccaagctgtaagtttaaacatgatcttactaactaactattctcatttaaattttcagagcttaaaaatggctgaaatcactcaca
acgatggatacgctaacaacttggaaatgaaat

Fluorescent microscopy

A mixed population of *hruEx154* and *hsf-1; hruEx154* animals was transferred to fresh OP50 plates and incubated at 9 °C for 24 h and at 20 °C for 12 h, respectively. Live worms were mounted onto 2.5% agar pads and anaesthetised using 0.1% sodium azide. Nomarski and fluorescent images were obtained using the fluorescence microscope BX51 and the equipped DP74 camera with cellSens software (OLYMPUS).

Dauer formation assay

The worms were prepared using the same protocol described above for the CID experiments. Approximately 100 eggs were transferred to OP50 bacterial lawns on fresh NGM plates and cultivated at 20 °C and 25 °C. The dauer formation of the strains was observed at 60 (25 °C), and 72 h (20 °C), respectively.

RNA isolation and qRT-PCR

The worms were prepared using the same protocol described above for the CID experiments. Approximately 5000 eggs were transferred to OP50 bacterial lawns on fresh NGM plates and cultivated at 9°C for 3 days. Hatched L1 animals were collected into 1.5 mL tube by washing with M9;

the supernatant was removed, and the worm pellets were stored at -80°C until RNA isolation. Total RNA was extracted from the worm pellets using NucleoSpin® RNA Plus kit (Takara-Bio), and cDNA was synthesised from the extracted RNA using SuperScript™ III Reverse Transcriptase (Invitrogen). Quantitative RT-PCR was performed using StepOne Plus Real-Time PCR System and PowerTrack SYBR Master Mix (Applied Biosystems). Gene expression was analysed using the comparative Ct method, and statistical analysis was performed by 2-way ANOVA using GraphPad Prism software. The combinations of primer sets used are listed in Supplementary Table 4.

Statistics analysis

No statistical method was used to predetermine sample size. No data were excluded from the analyses. The experiments were not randomized. For the ageing experiment, the worm strains were blinded. In other experiments, the worm strains were not blinded. Kaplan-Meier statistics was used to analyse the ageing experiment. *P*-value < 0.05 is considered significant.
